# Endophytes in *Cannabis sativa*: Identifying and Characterizing Microbes with Beneficial and Detrimental Effects on Plant Health

**DOI:** 10.3390/plants14081247

**Published:** 2025-04-19

**Authors:** Liam Buirs, Zamir K. Punja

**Affiliations:** 1 Pure Sunfarms Corp., Delta, BC V4K 3N3, Canada; lbuirs@puresunfarms.com; 2 Department of Biological Sciences, Simon Fraser University, Burnaby, BC V5A 1S6, Canada

**Keywords:** plant microbiome, biological control, soil microbes, plant pathogens, microbial diversity

## Abstract

The roles of endophytes in *Cannabis sativa* (cannabis, hemp) remain poorly explored. While in vitro studies suggest that there can be several benefits, such as plant growth promotion and protection against pathogens, more in planta studies are needed. This review summarizes the bacterial and fungal endophytes previously reported in tissues of *C. sativa* and discusses the factors influencing their presence, as well as their potential beneficial and detrimental effects. Using genome sequencing and culture-based approaches, we describe the microbial diversity in hydroponically cultivated cannabis plants at several developmental stages. These include mother plants, cuttings, vegetative and flowering plants, and tissue-cultured plantlets. Microbes that were present include fungal, yeast, and bacterial endophytes found in roots, stems, leaves, inflorescences, and seeds. These may have originated from the growing substrate or be transmitted through vegetative propagation. Notable endophytes included *Rhizophagus irregularis* (a mycorrhizal fungus), *Penicillium chrysogenum* (an antibiotic producer), and various endophytic yeast species not previously described in *C. sativa*. Endophytes representing potential plant pathogens, such as *Fusarium oxysporum*, are also present within cannabis tissues, which can negatively impact plant health. Using scanning electron microscopy, we observed that fungal propagules are present within pith parenchyma cells and xylem vessel elements in stem tissues, illustrating for the first time the in situ localization and distribution of endophytes in cannabis vascular tissues. The mechanism of spread through xylem vessels likely contributes to the spread of endophytes within cannabis and hemp plants. Further research is required to validate the roles of endophytes in cannabis and hemp plants grown under commercial production conditions.

## 1. Introduction

*Cannabis sativa* L., hereafter referred to as cannabis (THC > 0.3%) and hemp (THC < 0.3%), is believed to have originated in western or central Asia [1,2] and has been utilized by humans for centuries. Archeological evidence suggests that cannabis cultivation dates back approximately 6000 years in China [3,4]. Since then, its cultivation has spread across Asia, the Middle East, Africa, and Europe through trade routes, leading to further domestication [2]. Cannabis is propagated vegetatively from mother (stock) plants or is grown from seeds; their growth and flowering requirements are described elsewhere [5,6,7,8]. Numerous previous studies have assessed the origins of microbial communities associated with cannabis plants, and they have been categorized based on the tissues from which they are recovered. Endophytes colonize plant tissues internally (endosphere), while epiphytes colonize niches externally, including roots (rhizosphere), foliage (phyllosphere), and flowers (anthosphere) [9,10,11,12,13,14,15]. A sub-group of these microbes can represent opportunistic pathogens [16,17,18]. The endophytic microbes in cannabis and hemp plants may give rise to commensal, mutualistic, or pathogenic relationships, all of which warrant further exploration. The endophytes in cannabis plants have not been well studied from an epidemiological perspective. For example, how do propagation methods and growing conditions influence the endophyte composition? Little is known about the persistence of endophytes through successive plant propagation cycles or the influence of externally applied microbes on endogenous microbial populations.

This review summarizes previous studies on bacterial and fungal endophytes observed in cannabis and hemp tissues and outlines the influence of tissue origin, geographic location, genotype, and growing substrate on these populations. The biocontrol and bio-stimulant properties of *C. sativa* endophytes are described, as well as the sub-set of pathogenic species that can potentially cause disease or reduce quality. In addition, we also describe the diversity of microbes residing within cannabis plants grown under greenhouse hydroponic conditions using culture-based and microbiome sequencing approaches. Lastly, we examined the pith parenchyma cells and xylem vessel elements of *C. sativa* to reveal the presence of endophytic propagules in situ.

## 2. Isolation and Identification of Endophytes in Cannabis and Hemp Plants

Endophytes are reported to be recovered from plant tissues following different surface sterilization and culturing methods [16,19,20,21,22]. Tissue segments treated with sodium hypochlorite and ethanol are placed on an agar medium, such as potato dextrose agar (PDA), with or without antibiotics, or on other agar media, resulting in the recovery of endophytic fungi, bacteria, and yeasts [19,22,23]. The choice of media can be selected to target specific microbial groups. However, it should be recognized that not all internal microbes are culturable [24]; this underscores the importance of complementary molecular sequencing approaches. The tissue sterilization step is critical to avoid culturing epiphytic microbes and to ensure that only internal microbes are recovered. After recovery, microbial identification using the PCR of the ITS region of ribosomal DNA for fungi and the 16S rRNA gene for bacteria is accompanied by sequencing and comparison with databases such as GenBank to confirm the species that are present [22,23]. Identification to the species level is important to develop a database that allows for comparisons to be made between different published studies. To determine the nonculturable microbial profiles, whole genome sequencing (microbiome analysis) can be used as described later in this review.

## 3. Recovery of Microbes from Cannabis and Hemp Plants

A wide range of fungal and bacterial species have been reported to be present internally within various tissues and organs of cannabis and hemp plants following surface sterilization (Table 1). These include samples obtained from outdoor-grown cannabis and hemp plants and from greenhouse-grown cannabis plants. The tissues and organs studied were seeds, roots, crowns/stems, leaves/petioles, and inflorescences. Many microbes were represented by pathogens, while others had known benefits, and the rest were unclassified.

The microbial species composition is influenced by the tissue source, such as roots, stems, petioles, leaves, flowers, and seeds, as well as by the geographic origin and growing conditions of the plant [16,17,18,19,21,22,25,26,27,29,30,31,32]. These are described in more detail below.

### 3.1. Effect of Geographic Origin and Lineage on Microbial Diversity

An analysis of the seed microbiome of 46 cannabis accessions represented by different genotypes and breeding lines from eight institutions or seed companies across various regions of Europe was conducted by Lobato et al. [25]. Members of the genus *Bacillus* were most abundant in surface-sterilized seeds (Table 1). In contrast, the genus *Staphylococcus* was the most dominant, and the species *S. epidermidis* and *S. haemolyticus* were present in the seeds and plantlets, respectively, of hemp cultivar ‘Futura 75’ grown in Italy [26]. Other genera present included *Bacillus*, *Paenibacillus*, and *Sphingomonas* (Table 1). A majority of the endophytes present in 15 genotypes of cannabis and hemp seeds and seedlings from field- and indoor-grown plants in western Canada were *Bacillus* and *Paenibacillus* species [21], with *P. mobilis* being consistently present in all genotypes (Table 1). Only a single fungal species—*Penicillium brevicompactum*—was recovered from surface-sterilized seeds. These studies illustrate the diversity of bacterial species that can be present in the seeds and seedlings of different genotypes of cannabis and hemp from different growing locations. A subset of the microbes can be readily passed through seeds from the mother plant or the growing environment to the next generation [21,33]. The mechanism by which this occurs is unknown but likely involves movement through the xylem tissues directly into the developing seed. The types of microbes present in the seeds can be influenced by numerous factors, as described by Nelson [33].

By comparing seed sources that included landraces, selected lines, cross hybrids, and inbred lines, Lobato et al. [25] reported that less domesticated genotypes (landraces and selected lines) exhibited higher bacterial diversity compared to more domesticated genotypes (cross hybrids and inbred lines); the latter had reduced diversity and a more homogeneous bacterial community. This suggests that breeding efforts and selection may have inadvertently reduced microbial diversity within seeds, an intriguing finding. The effect of the ploidy level (triploid vs. diploid) of hemp on naturally occurring seed-derived endophytes was reported by Srivastava et al. [34]. Triploid plants hosted lower bacterial and fungal diversity than diploid plants following artificial inoculation, the basis for which is not known. Interactions between the cultivar and the plant growth stage that affected the microbial abundance in the rhizosphere, endorhizosphere, and phyllosphere at four growth stages—from propagation to late flowering—of indoor-grown cannabis plants were described by Comeau et al. [13]. The findings revealed that significantly different microbial communities were present across cultivars and that each compartment harbored distinct microbial communities, which was variable over the growth stages. The rhizosphere and endorhizosphere exhibited the highest diversity of fungal communities, and lower levels were present in the phyllosphere. Fungal and bacterial community richness and uniformity increased as the plants matured; e.g., specific fungal orders were present in each cultivar at the late flowering stage. In three industrial hemp cultivars grown in Quebec, the bacterial endophytes were reported to be uniformly distributed across cultivars, but the fungal endophyte distribution was influenced by the cultivar, and certain fungal species were specific to certain hemp cultivars [20]. These findings illustrate the potential influence of genetic backgrounds, cultivars, and growth stages on the microbial composition of cannabis and hemp plants, making the interpretation of these findings complex due to the many interacting plant variables that may influence endophyte composition. They also indicate the variability of the endophyte distribution in different studies, making comparisons difficult in some cases.

In a study exploring how both cultivar and soil type influence the cannabis endophyte profile, it was observed that bacterial species abundance varied between three cultivars grown in two different soils (hemp field topsoil and vineyard soil in Alberta and British Columbia, respectively) [35]. A core microbiome of bacterial endophytes, dominated by Gammaproteobacteria and Bacilli, was observed across all soil types and cannabis cultivars. This suggests that certain seed-borne endophytes are inherited and persist regardless of the presence or absence of soil microorganisms. However, in cannabis seedlings, the substrate microbial profile significantly influenced the diversity and abundance of endophytic bacteria. These findings suggest that the uptake of endophytes from the growing medium can be important during the early stages of plant growth.

### 3.2. Effect of Growing Substrate on Microbial Diversity

A comparison of the endophyte profiles of cannabis plants grown in an organic mixture comprising soil and composted plant waste materials with those grown hydroponically in rockwool and cocofibre showed that greater fungal diversity was present in the former [18,36]. Stem samples from organically grown plants harbored 17 species of fungi from 11 genera, with *Penicillium*, *Fusarium*, *Aspergillus*, *Chaetomium*, and *Trichoderma* being the most dominant. In contrast, stems from hydroponically grown plants yielded eight fungal species from six genera [17]. In addition, the organic substrate contained ~158 X more fungal colony forming units (CFUs) and ~1360 X greater bacterial CFUs compared to rockwool, suggesting that the higher endophyte diversity was correlated with higher microbial diversity in the growing substrate [18]. Similarly, cannabis seedlings grown in microbe-rich versus sterilized soils displayed a higher abundance and diversity of bacterial endophytes [35]. Betaproteobacteria were more prevalent in sterilized soil, while Gammaproteobacteria were enriched in non-sterilized conditions. These findings emphasize the role of the growing substrate on influencing endophyte distributions in cannabis plants.

### 3.3. Influence of Plant Organ Tissues on Microbial Diversity

A comparison of petiole, leaf, and seed tissues of three industrial hemp cultivars grown in Quebec showed that up to 18 bacterial and 13 fungal genera of endophytes were present, with *Pseudomonas*, *Pantoea*, *Bacillus*, *Alternaria*, and *Cochliobolus* found to be the most abundant [20]. Petiole tissues contained the most bacteria (67%), followed by leaves (19%) and seeds (14%), while most fungal isolates were obtained from leaves (70%), followed by petioles (26%) and seeds (4%). Gautam et al. [19] examined the leaves, stems, and petioles of cannabis plants grown outdoors and reported that endophytic fungi were recovered from 75.4% of the tissues. The frequencies were highest in stems (84.9%), followed by leaves (82.41%), and lowest in petioles (59.8%). Eight fungal genera that included 12 species were isolated (Table 1). *Aspergillus* was the most frequently recovered genus, followed by *Penicillium* spp. (*P. chrysogenum* and *P. citrinum*). The remaining genera included plant pathogens (Table 1). This study highlights the potential for undesirable endophytes being present in cannabis plants, since *Aspergillus* and *Penicillium* are both mycotoxin producers and are of concern if present in cannabis inflorescences [14]. Stem and petiole tissues appear to contain the highest levels of endophytes among above-ground tissues, consistent with their proposed spread through the xylem tissues.

Four Chinese hemp cultivars grown indoors in soil showed the highest endophyte diversity in roots, with moderate diversity in leaves and stems, and lowest in flowers [37]. Variations in microbial profiles were more strongly influenced by the soil microbial profile and the tissue compartments (roots, stems, and flowers) than by the cannabis genotype. The predominant bacterial genera included *Rhizobium*, *Pseudomonas*, *Planococcus*, *Bacillus*, and *Sphingomonas*, while the predominant fungal genera were *Clitopilus*, *Plectosphaerella*, *Alternaria*, *Hypocrea*, and *Mortierella*, each of which exhibited significant compartment-specific distributions. A significant portion of the endophytes was similar to those present in soil, highlighting the influence of the soil microflora on the endophytic communities within hemp plants. These studies show that variability in endophyte type and incidence is influenced more by the tissue source and growing substrate than by the cultivar grown. Roots exposed to microbially active soil would be expected to harbor the highest levels of endophytes.

### 3.4. Impact of Endophytes in the Root Zone on Plant Development

The occurrence of endophytes in the endorhizosphere can be influenced by the epiphytic populations in the rhizosphere, which are classified based on their interaction with the host plant as facultative, obligate, or passive [38,39]. Epiphytic microbes can provide benefits to the plant [40,41,42,43,44,45,46] and include plant growth-promoting rhizobacteria (PGPR) and mycorrhizal fungi, which can form unique tripartite interactions in this niche [47]. These epiphytes can also affect the proportion that becomes endophytic. The specific roles of these endophytic microbes in influencing cannabis growth need to be determined. The microbes originating as endophytes, when recovered and re-applied to cannabis or hemp plants, can influence their growth. For example, applications of biocontrol agents that include endophytic species externally to the root system have been shown to enhance growth and reduce pathogen development on cannabis plants [8,22]. Similarly, applications of multiple formulations of bacterial and fungal species to growing substrates have improved the growth and yield of cannabis plants [28,34,48,49,50] (Table 2 and Table 3), confirming what is reported in many other plant species [23,51,52,53]. These studies point to the benefits provided by certain groups of endophytes when applied to the root zone on promoting cannabis growth. The underlying mechanisms by which biocontrol or bio-stimulant activity by endophytes can improve cannabis growth have been researched. Some of these are described in more detail below.

## 4. Influence of Bacterial and Fungal Endophytes on Plant Growth

Some endophytes have demonstrated potential to enhance cannabis plant growth through mechanisms involving biocontrol and bio-stimulation, resulting in reduced pathogen severity, improved plant health, and increased production of secondary metabolites. However, comprehensive and integrated approaches to optimize the applications of cannabis endophyte populations to consistently enhance plant growth have yet to be developed [62]. The results from specific studies that involve one to several endophyte species provide a starting point toward achieving this goal and are described in more detail below.

### 4.1. Beneficial Bacterial Endophytes

Commonly reported bacterial endophytes in cannabis and hemp plants include species of *Bacillus*, *Paenibacillus*, *Pseudomonas*, *Pantoea*, and *Enterobacter* [21,50,63,64]. These endophytic bacteria can exhibit beneficial properties, such as antibiotic production, the induction of host defense responses, growth promotion, competition, parasitism, and quorum signal interference [30,39,65,66]. Bacterial endophytes may improve the overall growth of cannabis and hemp plants through various mechanisms. These bacterial endophytes may produce phytohormones, stimulate the production of various phytochemicals, and enhance nutrient availability to increase plant growth or vigor [25,48,49,54,58]. They are also reported to protect the plants from pathogens through various forms of biological control and induced systemic resistance [20,21,23,27,55,56,57,59,63]. The published reports on endophytic bacterial species that have demonstrated bio-stimulant or biological control properties on cannabis or hemp plants are summarized in Table 2.

There are three bacterial species that are reported to be endophytes of cannabis plants which are currently registered as bio-fungicides in Canada and can be used as biological control agents. The first is *Streptomyces lydicus* WYEC 108 (in Actinovate^®^ SP or CannaPM), which can provide biological control of *Botrytis cinerea*, *Sphaerotheca macularis*, and *Pythium* spp. The second bacterial species is *Bacillus amyloliquefaciens* D747 (in Double Nickel^®^ LC/55), which can provide biological control of *B. cinerea*, *Sclerotinia sclerotiorum*, *Golovinomyces cichoracearum*, and *Podosphaera macularis.* The third is *Bacillus mycoides* isolate J (in LifeGard WG), which can provide biological control of *B. cinerea* and *S. sclerotiorum*. More information on these registered products can be found at Health Canada—Pesticide Label Search (https://pr-rp.hc-sc.gc.ca/ls-re/index-eng.php (accessed on 18 December 2024). It should be noted that current regulations surrounding the use of microbial species as biological control agents on cannabis plants require extensive assessments for non-target effects and must satisfy regulatory standards set out by governmental agencies in order to be used during commercial production. Currently, very few endophytic bacterial species shown in Table 2 have fulfilled these requirements.

### 4.2. Beneficial Fungal Endophytes

Commonly reported fungal endophytes of cannabis and hemp plants include *Aspergillus*, *Cladosporium*, *Alternaria*, *Fusarium*, *Penicillium*, *Trichoderma*, *Paecilomyces*, and *Chaetomium* [8,16,19,22,64]. A number of these endophytes are reported to confer bio-stimulant effects on cannabis plants through various mechanisms, including phytohormone or secondary metabolite production [28,67], thus enhancing vigor, yield, and cannabinoid levels [28,60]. They may also enhance defense responses via the activation of defense pathways and the production or solubilization of compounds that enhance plant fitness [23,39,60,65,66,68,69]. Also, competition, antibiotic production, and mycoparasitism may be involved [16,22,39,70,71,72]. The endophytic fungal species that have demonstrated bio-stimulant or biological control properties on cannabis and hemp plants are summarized in Table 3.

There are a number of fungi reported to be endophytes which are registered as bio-fungicides in Canada and used as biological control agents. These include *Trichoderma asperellum* T34 (in Asperello T34), *Gliocladium catenulatum* J1446 (in PreStop), *T. harzianum* KRL-AG2 and *T. virens* G-41 (in RootShield^®^), *T. harzianum* T22 (in Trianum P), and *T. asperellum* ICC 012 and *T. gamsii* ICC 080 (in Foretryx^TM^). Collectively, these products can provide biological control of species of *Pythium*, *Fusarium*, *Phytophthora*, *Rhizoctonia*, *Verticillium*, and *B. cinerea*. The details of these registered products can be found at Health Canada—Pesticide Label Search (https://pr-rp.hc-sc.gc.ca/ls-re/index-eng.php (accessed on 18 December 2024)). As noted previously for bacterial endophytes, current regulations surrounding the use of microbial species as biological control agents on cannabis plants require extensive assessments for non-target effects and must satisfy regulatory standards set out by governmental agencies before they can be used during commercial production.

The application of *Beauveria bassiana* (strain NATURALIS) and *Metarhizium brunneum* (strain BIPESCO5) to the growing medium of pepper seedlings was reported to reduce the development of Fusarium crown and root rot through endophytic colonization by these biocontrol agents [73]. These two entomopathogenic fungi have been previously recovered from cannabis tissues [17], possibly due to their usage in crops as registered pest control products. However, these two species still require efficacy studies to show whether they have utility as beneficial endophytes on cannabis plants. There are a large number of variables that can influence the extent to which an introduced endophytic fungal species can colonize the growth substrate and gain access to the root system to establish a successful relationship (engraftment) [74]. These include microbial traits, host traits, competition or synergy with pre-existing microbes in the substrate, and the influence of the surrounding environment. They have been eloquently described by Poppeliers et al. [74] and need to be evaluated in the context of cannabis plant growth. For example, Moshe et al. [75] studied the persistence of a *Bacillus* biocontrol agent and its effect on the native microbiomes in the rhizosphere of pathogen-inoculated cucumber plants. They demonstrated that the amendment could significantly alter local microbiomes and concluded that optimizing amendment regimes or selecting strains with higher rhizosphere competence could enhance the efficacy of biocontrol agents. Similar studies are currently needed for cannabis and hemp.

### 4.3. Detrimental Bacterial Endophytes

Reports of bacterial species that can be endophytic and also cause detrimental effects on cannabis and hemp plants are few. While some pathogenic species have been described, which include *Agrobacterium tumefaciens* causing crown gall on cannabis [76] and *Pseudomonas syringae* causing blight on hemp plants [77], the remaining species shown in Table 2 remain uncharacterized. However, to achieve commercial utility with bacterial species to meet regulatory guidelines, extensive assessments for non-target effects, as set out by governmental agencies, are required. As such, while many of the findings are scientifically interesting and show effects from in vitro studies, these bacteria are required to meet the criteria needed for regulatory approval before they can be utilized as biological control agents or bio-stimulants in practice.

### 4.4. Detrimental Fungal Endophytes

A number of endophytes of cannabis plants include plant pathogenic fungi, such as *Fusarium* spp. and *B. cinerea*, which cause damping off, leaf blight, and bud rot [14,16,17,78]. For plants growing outdoors, a range of fungi, including *Alternaria*, *Aspergillus*, *Phoma*, *Colletotrichum*, *Cladosporium*, and *Curvularia*, have been recovered as endophytes [19,29], some of which are known to be plant pathogens. In previous studies, the recovery and inclusion of endophytic plant pathogenic species during in vitro testing for antagonism against other fungi, as described by Priyashantha et al. [79] and Kusari et al. [16], are counter-intuitive, and they should be excluded from further testing. These fungi would not be considered to be useful for applications to cannabis plants and would fail to meet regulatory requirements. Such pathogenic fungi may also be present on cannabis seeds or in vegetative cuttings derived from mother plants [8,18,80]. Others may originate as contaminants from the growing substrate or external environment [17]. A number of endophytic fungal pathogens, including *Fusarium* spp. and *B. cinerea*, can remain latent until environmental conditions trigger them to become pathogenic [78,81]. Further research is needed to determine whether some detrimental fungal endophytes, such as *Aspergillus*, *Cladosporium*, *Fusarium*, and *Penicillium*, may contribute to mycotoxin accumulation in cannabis tissues or colonize inflorescence tissues, thereby reducing their quality [14,17,82].

To reduce the risks associated with microbes persisting inside or on cannabis inflorescences, gamma and electron beam irradiation may be used as these methods are effective in reducing microbial populations below the thresholds set by regulatory agencies [83,84,85,86]. Such approaches may be employed if microbial levels exceed regulatory limits or if zero tolerance is required, e.g., for medical cannabis patients [14]. To minimize the risk of inflorescence tissues containing endophytic microbes, sources of propagative materials, including seeds and cuttings, should be tested for pathogenic fungal presence. Tissue culture-derived starting materials may also reduce the incidence of internal tissue colonization by microbes [87,88,89,90]. Tissue-cultured plantlets may, however, become rapidly colonized by endophytic fungi and bacteria once reintroduced into the growing environment, which can originate from the substrate or surrounding environment. Therefore, it is important to consider the potential factors that may contribute to an increase in microbial levels during the cultivation cycle. The interactions with and complexity of the microbial populations associated with cannabis plants (microbiome) remain poorly understood. Factors contributing to microbial colonization through the cultivation cycle, in addition to applications of preventative inoculations to proactively establish beneficial endophytic communities, are not well understood and are likely to be impacted by numerous factors, as described by Poppeliers et al. [74] and Navi et al. [91].

## 5. Analysis of Cannabis Endophytes Using Whole Genome Sequencing

**Figure 1:**
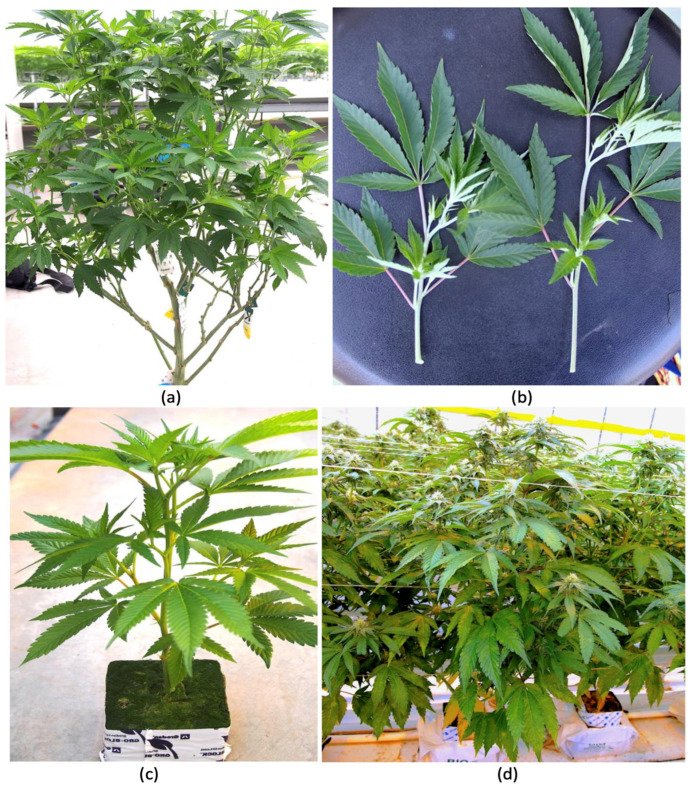
The different stages of growth of greenhouse-cultivated cannabis plants from which tissue samples were obtained for microbiome and microscopic analyses. (**a**) Mother (stock) plants approximately 3 months of age. (**b**) Vegetative cuttings taken from mother plants used for propagation. (**c**) A vegetative plant approximately 4 weeks following the rooting of cuttings. (**d**) Flowering plants approximately 4 weeks into flowering.

**Figure 2:**
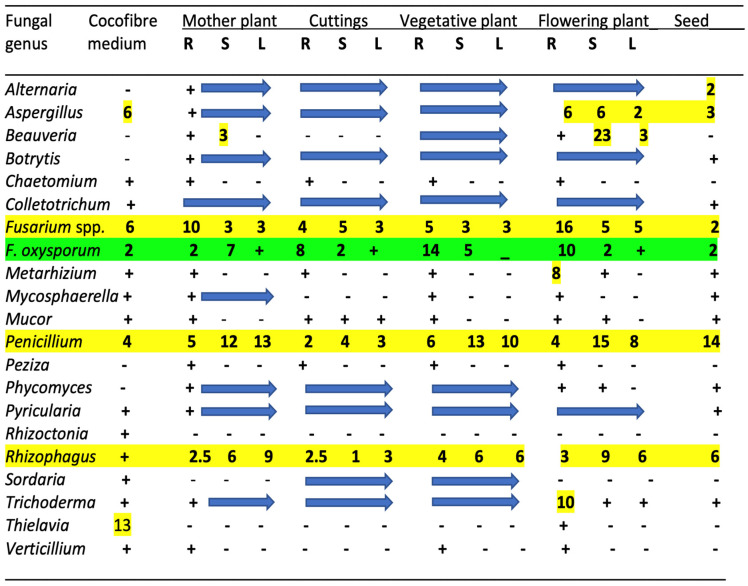
Genera of endophytic fungi identified in cannabis tissues at different stages of development and in the growing substrate as determined by whole genome sequencing. Numbers represent the percentage of the total reads of the specific genus relative to the total fungal reads in the tissue sample. The “+” and the blue arrows indicate a value of up to 1% of the total reads. Values over 1% are highlighted in yellow or green.

To determine the prevalence of endophytic fungi and yeasts, greenhouse-cultivated cannabis plants at various stages of development were studied. Mother plants (varying in age from 2 months to 6 months) (Figure 1a), cuttings derived from them (Figure 1b), and vegetative (4-week-old) (Figure 1c) and flowering plants (4-8 weeks old) (Figure 1d) grown as described elsewhere [8,81] were obtained from up to 10 cannabis genotypes for microbiome analysis. None of these plants displayed any symptoms of disease. Root, stem, and leaf samples were collected randomly from these plants and sterilized using 70% ethanol and 10% bleach (containing 0.525% NaOCl) in various combinations of time, ranging from 30 sec to 3 min, depending on the tissue source. Also, three seed samples were included for analysis: hemp seeds from a supplier in Colorado, high-THC cannabis seeds from a greenhouse producer in British Columbia, and seeds from an online supplier. After tissue samples were harvested, they were flash-frozen in liquid nitrogen and stored at −80 °C. Samples were homogenized into a fine powder using a mortar and pestle under liquid nitrogen to ensure thorough cell disruption. Genomic DNA was extracted using the Qiagen DNeasy Plant Mini Kit (Qiagen, Inc., Toronto, ON, Canada) following manufacturer’s protocol, with additional steps to optimize the yield and purity tissues containing high levels of secondary metabolites. DNA quality and quantity were assessed using a NanoDrop spectrophotometer, while DNA integrity was confirmed through electrophoresis on a 1% agarose gel. DNA samples were sent to Medicinal Genomics (Beverly, MA, USA) (https://medicinalgenomics.com/genomic-services/ (accessed on 10 March 2024)) for high-throughput metagenomic sequencing (Next Generation Sequencing). Both fungal and yeast communities were profiled to capture the diversity of these microbial communities. Approximately 60 samples were assayed in total. The data from the same tissue types were combined for analysis. Sequencing data were saved as FASTA files containing adapter-trimmed high-quality reads. Reads were classified using a metagenomics classification algorithm that assigned a taxonomic identification to each sequencing read and estimated the abundance of each species in the sample. Taxonomic outputs were visualized using Krona plots to enable interactive and hierarchical visualization of microbial diversity. This approach facilitated a detailed exploration of microbial community structure and differences across plant parts and growth stages. The data presented in Figure 2 represent the percentage of total reads of the specific genus relative to total fungal reads in the tissue samples from specific plant growth stages. To confirm the presence of certain culturable endophytes, surface-sterilized stem pieces were incubated on potato dextrose agar containing 130 mg/L of streptomycin sulfate for up to 2 weeks to recover fungal endophytes [17].

The results from the microbiome analysis of all cannabis tissue sources are presented for fungal genera in Figure 2. Only the genus names are presented except for select species that are discussed in more detail below. These microbes represent reads that were >1% of the total reads for each microbial category. Genera with significantly higher reads are also identified in the figures. For fungal endophytes, 20 genera are shown in Figure 2, with the most abundant being (in decreasing order) *Fusarium*, *Penicillium*, *Rhizophagus*, and *Aspergillus*. These genera were present in all tissue types and plant sources, including seeds (Figure 2). The data for the presence of bacterial endophytic species are not presented.

The remaining genera present in all tissue types at a lower frequency (ca. 1%) included *Alternaria*, *Botrytis*, *Colletotrichum*, *Phycomyces*, *Pyricularia*, and *Trichoderma*. Other genera that were present in the roots of flowering plants were *Beauveria* and *Metarhizium*, which are biocontrol agents that were applied during cultivation and likely became established in the treated plants. While the plant pathogens *Rhizoctonia*, *Mycosphaerella*, and *Verticillium* were present in the growing medium, they were detected infrequently in host tissues. Finally, the genus *Thielavia*, a heat-tolerant saprophytic fungus capable of degrading cellulytic substrates [92], was present in the growing medium but was only detected in the roots of one sample.

Specific fungal genera present in the growing substrate were strongly correlated with those found in cannabis tissues, suggesting that this was a likely source of many of the fungi. These included *Fusarium*, *Penicillium*, *Aspergillus*, *Colletotrichum*, *Pyricularia*, and *Trichoderma* (Figure 2). Of note is that many of these fungi are plant pathogens. However, some fungi present in plant tissues but not present in the substrate, such as *Alternaria*, *Beauveria*, *Botrytis*, *Peziza*, and *Phycomyces*, likely originated from external environmental sources, as well as from the air, and subsequently became established in the plants. Previous air sampling studies conducted in greenhouses have shown that many of these fungi produce air-borne spores and can be detected on agar settling plates [17,32,81]. Exposed pruning sites and cut surfaces on stems are prone to colonization by various fungi in cannabis cultivation facilities [17,81]. Other genera that were found sporadically in the tissues included *Chaetomium*, *Mycosphaerella*, *Mucor*, *Peziza*, and *Sordaria*. These results demonstrate the dynamic nature of fungal endophyte establishment in cannabis plants under greenhouse conditions that can originate both from the growing substrate and from various external sources. Many of these fungi cannot be considered as beneficial because they include plant pathogens, and others are designated as saprophytes. In addition, the findings indicate that a range of endophytic fungi are inherently present in mother plants, and they can be carried over in cuttings taken from these plants which are used for vegetative propagation to initiate a new cycle of crop production. Such horizontal transmission indicates that resident microbes in cannabis cuttings originating from mother plants or seeds may already contain a resident microbial background (load). This should be considered when evaluating the effects of externally applied microbes to determine the impact on plant growth, as the potential influence of existing fungal (and bacterial) species already present internally should be recognized. The analysis of a limited number of seed samples showed that they may contain up to 12 genera of fungi. These included *Alternaria*, *Botrytis*, *Colletotrichum*, *Fusarium*, *Mycosphaerella*, *Mucor*, *Pyricularia*, and *Phycomyces*. The source of these fungi cannot be ascertained as the seeds were obtained as samples that did not originate from the plants analyzed in this study. Seeds are known to harbor a range of endophytic fungal and bacterial species [21,33,35].

To potentially reduce the background microbial load within a commercial cultivation operation, using plants initiated from meristem tip cultures is a proven approach [88]. In addition, the experimental application of a systemic fungicide, such as ‘Luna Privilege’ (active ingredient fluopyram), to mother plants strictly in a research laboratory setting was previously shown to promote the success rate of propagation in tissue cultures by eliminating the most prevalent endophyte contaminants [88]. These included species of *Penicillium*, *Aspergillus*, *Fusarium*, *Chaetomium*, and other fungi that are found in cannabis plants. While fungicides such as ‘Luna Privilege’ are not currently registered for use on cannabis, our previous observations suggest that reducing the existing endophytes can allow for the studies of plant performance following the application of supplemental endophytic species to be more meaningful. Similarly, seed treatments that include bleach or fungicides should reduce the frequency of seed-borne endophytes and epiphytes. The use of heat treatment of seeds or potentially of cannabis cuttings may also be approaches to reduce the carry-over effects of detrimental endophytes.

**Figure 3:**
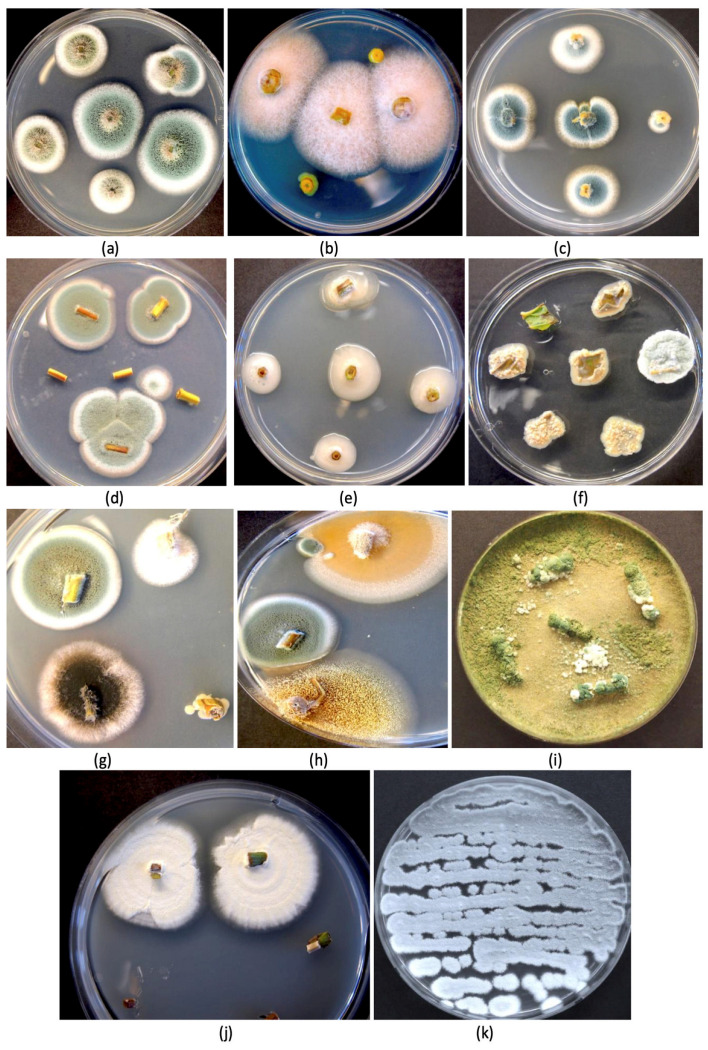
Recovery of a range of fungi from the stem, leaf, and petiole tissues of cannabis mother plants. All tissues were surface-sterilized before plating onto potato dextrose agar containing 140 mg/L of streptomycin sulfate to inhibit bacterial growth. (**a**) Two different colony types of *Penicillium* spp. (**b**) *Fusarium oxysporum* colonies. (**c**) Colonies of *Penicillium chrysogenum*. (**d**) Colonies of *Penicillium* sp. (**e**) Unidentified bacterial colonies. (**f**) Yeast colonies from leaf segments. (**g**,**h**) A range of colonies of different fungi, including *Penicillium* and *Aspergillus* (black and yellow colonies). (**i**) Recovery of *Trichoderma harzianum* from petiole segments. (**j**) Colonies of *Beauveria bassiana*. (**k**) Streaked culture of *P. chrysogenum*. All photos are of 9 cm diameter Petri dishes.

Surface-sterilized stem and leaf tissues from mother plants and flowering plants of cannabis incubated on an agar medium containing streptomycin sulfate (to inhibit the growth of bacteria) confirmed the presence of a range of endophytic fungal genera in these tissues (Figure 3). These included *Aspergillus* and *Penicillium* (Figure 3a), *Fusarium* (Figure 3b), *Penicillium* (Figure 3c,d), yeasts (Figure 3e), bacteria (Figure 3f), and a mix of *Aspergillus*, *Penicillium*, and other unidentified fungi (Figure 3g,h). The recovery of culturable fungi correlated well with the results from the microbiome analysis (Figure 2). Culturing methods were previously used to isolate and identify these species from various cannabis tissues [17,32,81].

An interesting finding in the present study was the ubiquitous presence of *Penicillium chrysogenum* in all 60 samples, including seeds, at detectable levels. In addition, *Rhizophagus irregularis* (former name *Glomus intraradices*) [93], an arbuscular mycorrhizal fungus, was also present in all tissue samples, including seeds (Figure 2). *Penicillium chrysogenum* produces the antibiotic penicillin [94], and its presence in all cannabis tissues of varying genotypes suggests that it has become adapted to this host and may be playing an important role, although additional studies are required to confirm this. This species was reported in a previous study to be present on cannabis inflorescences as a contaminant during the drying process [32]. A colony of *P. chrysogenum* isolated from cannabis stems is shown in Figure 3k. While many *Penicillium* spp. can cause post-harvest problems in cannabis inflorescences, there are a number of benefits that they confer as endophytes in numerous plants. *Penicillium chrysogenum*, reclassified as *P. rubens* [94], is known to produce the antibiotic penicillin [94,95,96] and has been recovered from the crowns and stem, petiole, and leaf tissues of cannabis plants [17,19,31]. In other crops, *P. chrysogenum* has been reported to confer various benefits, including biological control of plant pathogens [97,98,99] and the enhancement of plant development [100,101,102]. Further studies to evaluate other positive attributes of *P. chrysogenum* on cannabis plants are warranted. The second most prevalent species was *P. citrinum*, which has also been reported to be present in cannabis stem and petiole tissues [17,19] and in cannabis inflorescences [32]. This species is reported to function as a beneficial endophyte as well, providing plant growth promotion and biological control properties [103,104,105]. Although both *Penicillium* species may confer benefits as endophytes, they have also been recovered as epiphytes contaminating cannabis inflorescences from both indoor and outdoor crops [81]. The production of antibiotic compounds, including penicillin and citrinin, by these species may raise concerns if they are perceived to affect the health of cannabis consumers and are produced in sufficient quantities to be of concern [14]. They would therefore need to meet the regulatory guidelines outlined previously in order to be used on cannabis plants.

A second interesting microbe detected in all tissues, including seeds, was the mycorrhizal fungus *R. irregularis*, whose origin may be the growing substrate or seeds and cuttings as the substrate was not applied to the plants utilized in this study. This species is known to promote plant development in several ways [106,107], and it can confer bio-stimulant properties on cannabis plants [28,61]. Mycorrhizal-based inoculants were reported to enhance phytocannabinoid production when applied to the roots of cannabis plants [28,61], as well as promote plant growth [60,61]. The role of this mycorrhizal species in cannabis plants awaits further investigation, including the type of propagule (spores and mycelium) that may be present inside stems and leaves. Future research on endophytes to enhance cannabis growth should prioritize these two fungi because of their prevalence in cannabis tissues and their demonstrated positive effects from the published literature on other plant species. They should also be able to fulfill regulatory requirements more readily.

**Figure 4:**
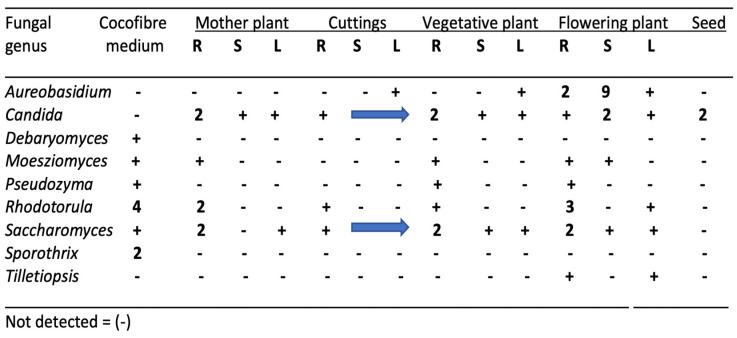
Genera of endophytic yeasts present in cannabis tissues and in the growing substrate as determined by whole genome sequencing. Numbers represent the percentage of total reads of the specific genus relative to the total fungal reads in the tissue sample. The “+” and the blue arrows indicate a value of up to 1%. Values over 1% are indicated.

The common genera of yeasts that were present in cannabis tissues are shown in Figure 4. The majority were present in the growing substrate from which they are presumed to have originated, but others were likely introduced by air-borne sources. These included *Aureobasidium*, *Candida*, and *Tilletiopsis*. In field-grown hemp inflorescences, *T. washingtonensis* was found to be commonly present [12]. Yeasts, in general, do not pose concerns in cannabis quality assessments unless the populations exceed the total yeast and mold levels set by regulatory agencies [32], or the presence of some genera such as *Candida* poses a health concern for susceptible individuals. There are no previous reports utilizing yeast species for biological control or bio-stimulant research on cannabis or hemp.

The microbiome data presented in this study shed light on the widespread nature of endophytic fungal and yeast species shown to be present in greenhouse-cultivated cannabis tissues, some of which are potential plant pathogens, while others may be saprophytic or incidental in nature. The growing substrate had a significant impact on the types and quantity of microbes in these tissues. Plants initiated from cuttings taken from mother plants containing endophytic species would likely also harbor a similar background of fungal and bacterial species. With the exception of *Beauveria*, *Mycosphaerella*, and *Sordaria*, which were present in mother plants at low levels and not detected in cuttings derived from them, the carry-over population of microbes was significant. Similar to the high levels of transmission of important plant pathogens, such as *F. oxysporum* [81] and hop latent viroid [108] in cuttings taken from diseased mother plants, cannabis cuttings appear to also harbor a significant proportion of endophytes that are derived from the mother plants. Avenues to remedy this background microbiome are discussed below.

## 6. The Endophytes in Cannabis Cuttings

**Figure 5:**
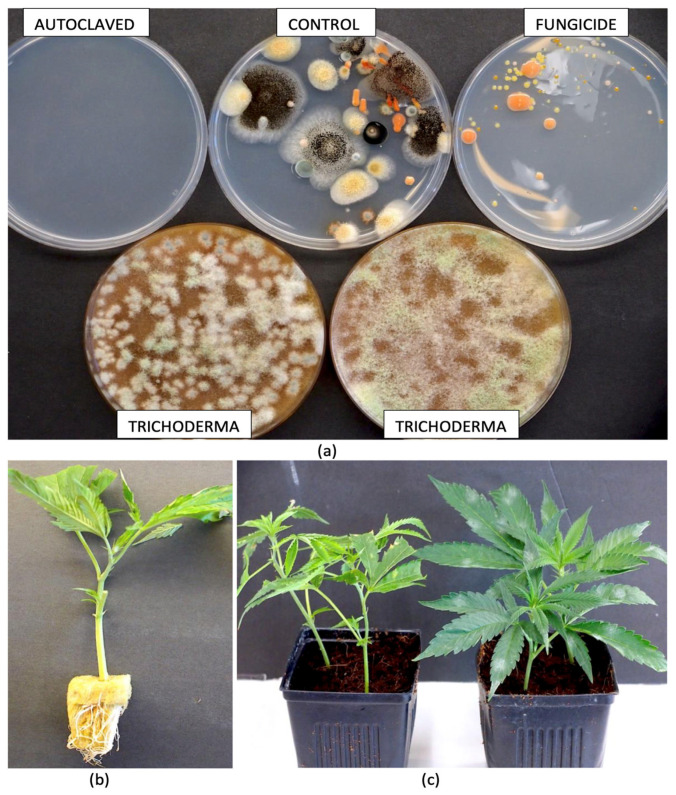
Treatments applied to the cocofibre growing substrate to alter the endophytes in cannabis cuttings. The substrate was autoclaved, received a drench of Luna fungicide, or was amended with formulations of *Trichoderma* found in Rootshield^®^ and Asperello^®^ after autoclaving. The substrates were then allowed to incubate under ambient laboratory conditions for 7 days before dilution plating was conducted to determine the background levels of mycoflora compared to the untreated control. In (**a**), the Petri dishes show microbial growth 5 days after dilution plating from each treatment. Note that there was no growth in the autoclaved substrate, while fungicide application reduced all fungal colonies compared to the control. In (**b**), rooted cuttings were inserted into the growing substrate following the treatments shown in (**a**) and then allowed to grow for 4 weeks before tissues were collected for microbiome analysis. (**c**) Comparison of cuttings inserted into the cocofibre substrate at the time the experiment was initiated (left) compared to after 4 weeks of growth (right). Root and stem tissues were collected, surface-sterilized, and prepared for microbiome analysis.

To determine the influence of substrate treatments on the subsequent detection of microbes in cannabis cuttings grown in treated growing media, the following treatments were experimentally applied to the moistened cocofibre growing substrate: autoclaved substrate (121 °C for 20 min), fungicide-drenched substrate (Luna), autoclaved substrate treated with *Trichoderma* products (Rootshield^®^ and Asperello^®^), and an untreated control. Following the treatments, the samples of growing substrate were incubated inside plastic bags for 7 days under ambient laboratory conditions (21 ± 2 °C). This was followed by dilution plating by suspending 1 gm in 9 mL of sterile distilled water and plating onto potato dextrose agar containing 130 mg/L of streptomycin sulfate. Developing colonies were rated after 5 days of incubation (Figure 5a). All substrates received 2-week-old rooted cuttings of cannabis genotype ‘PD’ seven days after treatment that were planted in 8 cm pots, placed under a 24 h photoperiod, and left to grow for 4 weeks (Figure 5b,c). Plants were watered as needed, and root and stem samples were collected from replicate plants in each treatment. The tissues were then surface-sterilized using a combination of bleach and ethanol and prepared for DNA extraction and microbiome analysis as described previously.

**Figure 6:**
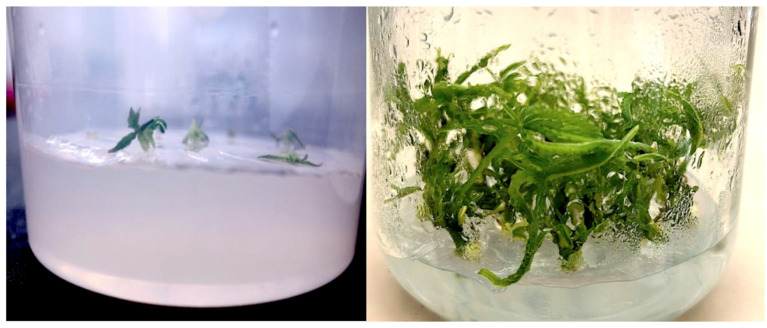
Tissue culture-derived plantlets from a meristem culture (left) and from a nodal culture (right) were assayed to determine the background microbes present. Tissue culture conditions were as described by Punja et al. [108], and leaf tissues were collected and processed for microbiome analysis as described in the methods section.

In addition, tissue-cultured plantlets derived from meristems and nodal explants as described by Punja et al. [108] (Figure 6) were grown for 6 weeks in culture vessels, introduced into the rockwool substrate, and placed in a growing environment under domes for 2 weeks; then, leaf tissues were collected for microbiome analysis as described previously. A comparison was made between the nodal- and meristem-derived plantlets for microbes present in the respective tissues.

**Figure 7:**
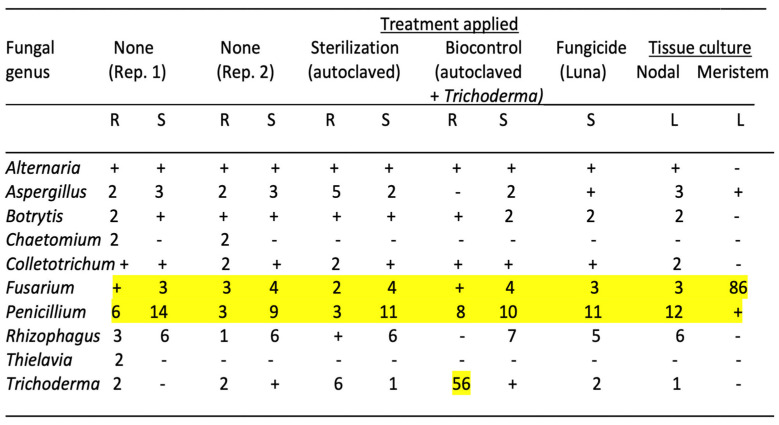
Effect of treatments applied to the cocofibre growing substrate on the resulting fungal microbiome detected in roots (R), stems (S) and leaves (L) of cannabis cuttings after a 4-week exposure to the treated substrate. Controls (no treatment) represent replicate samples (Rep. 1 and Rep. 2). Numbers represent the percentage of total reads of the specific genus relative to the total fungal reads in the tissue sample. The “+”indicate a value of up to 1% of the total reads. Values over 1% are highlighted in yellow.

The genera of fungi present in cannabis cuttings subjected to various treatments of the growing substrate or following tissue culture conditions are shown in Figure 7. In the untreated control, 10 fungal genera were present. Autoclaving the growing substrate prior to adding the rooted cuttings did not alter the composition of the fungi in root and stem tissues, suggesting that the pre-existing fungi were not altered. However, while the background fungal composition of cuttings receiving *Trichoderma* treatment was similar to the untreated control, there was a significant increase in the frequency of *Trichoderma* in the roots to a total of 56% of all reads (Figure 7). The treatment of the growing substrate with the Luna fungicide did not alter the composition of the fungi in roots and stems, suggesting that the pre-existing fungi were not altered. Overall, after comparing tissue culture-derived leaf tissues, those originating from nodal stem explants were not different in the fungal endophytic composition compared to the untreated control cuttings. In contrast, while those from meristems were initially devoid of all microbes except for *Aspergillus* and *Penicillium*, there was a significant increase in the presence of *Fusarium*, totaling 86% of all reads (Figure 7). The mycorrhizal fungus *Rhizophagus* was absent in meristem-derived leaf tissues but was present in those derived from nodal explants.

The results from this study highlight the persistence of the fungal endophytes in cuttings despite being exposed to a microbially active growing substrate. Autoclaving the substrate to eliminate all microbes did not alter the pre-existing fungal composition within the cuttings, which was inherited from the source mother plant. However, in the sterilized growing substrate amended with fast-growing *Trichoderma* spp., a significant endophytic component of root tissues was represented by this species. Nodal explants continued to harbor the background fungal endophyte composition, but the meristem culture eliminated almost all of the background fungal species. However, a rapid re-colonization of roots likely through air-borne spore contamination by *Fusarium* demonstrated the rapidity through which this microbial void in meristem-derived shoots could be replaced once plantlets were introduced into an exposed growing environment. These findings illustrate the importance of introducing potentially beneficial microbes that can establish as endophytes in the early stages of rooting of cannabis cuttings, despite the persistence of the pre-existing microbes in these tissues, which could only be eliminated through meristem tip culture. fungicide treatment of the growing substrate did not influence the pre-existing microbes in the cuttings. Meristem-derived plantlets may be more susceptible to rapid colonization by pathogenic fungi, perhaps as an outcome of the elimination of most pre-existing fungal endophytes. This hypothesis deserves further experimentation as it indirectly points to pre-existing fungal endophytes providing protection against invasion by pathogens such as *Fusarium*. It could also be the result of meristem tissue culture conditions making plantlets more susceptible to pathogens, but this was not seen in the nodal-derived plantlets grown under the same conditions.

## 7. Determining Endophyte Presence in Cannabis Tissues Using Scanning Electron Microscopy

**Figure 8:**
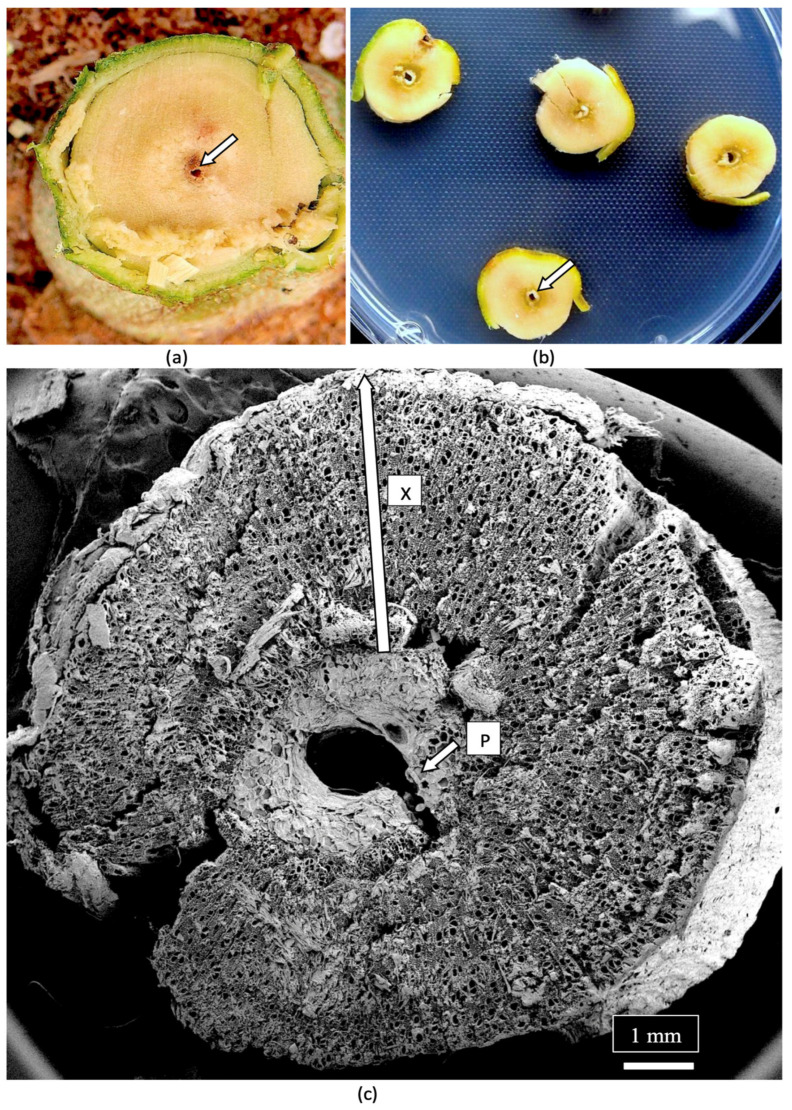
(**a**) Cross-sectional view of the main stem of a 3-month-old cannabis mother plant that shows the central pith (arrow). (**b**) Cross-sectional views of the side stems on a mother plant showing the central pith regions (arrow). (**c**) A scanning electron microscopic view of a section through a side stem as shown in (**b**). The central pith (P) is surrounded by a ring of xylem parenchyma cells that appear white. The largest area of the stem is occupied by the xylem (X) tissue that extends from the region of the pith cells to the outer ring of the phloem and epidermal tissues (arow).

The composition of the stems of mother plants and of the cuttings derived from them was studied to determine the tissue types and tissue arrangement commonly found in cannabis plants that have not been previously studied. The objective was to visualize and describe the tissue types and to determine if any endophytes could be discerned. The stems of mother plants were harvested from 3-month-old plants, and cross sections and longitudinal sections were prepared by hand sectioning for scanning electron microscopy, as described by Punja et al. [109]. The cross section of a stem of a mother plate revealed the hollow central pith (Figure 8a,b), which when examined under the scanning electron microscope revealed a ring of pith parenchyma cells surrounding the pith opening (Figure 8c). The remaining tissues surrounding the pith were cells representing the xylem tissue.

**Figure 9:**
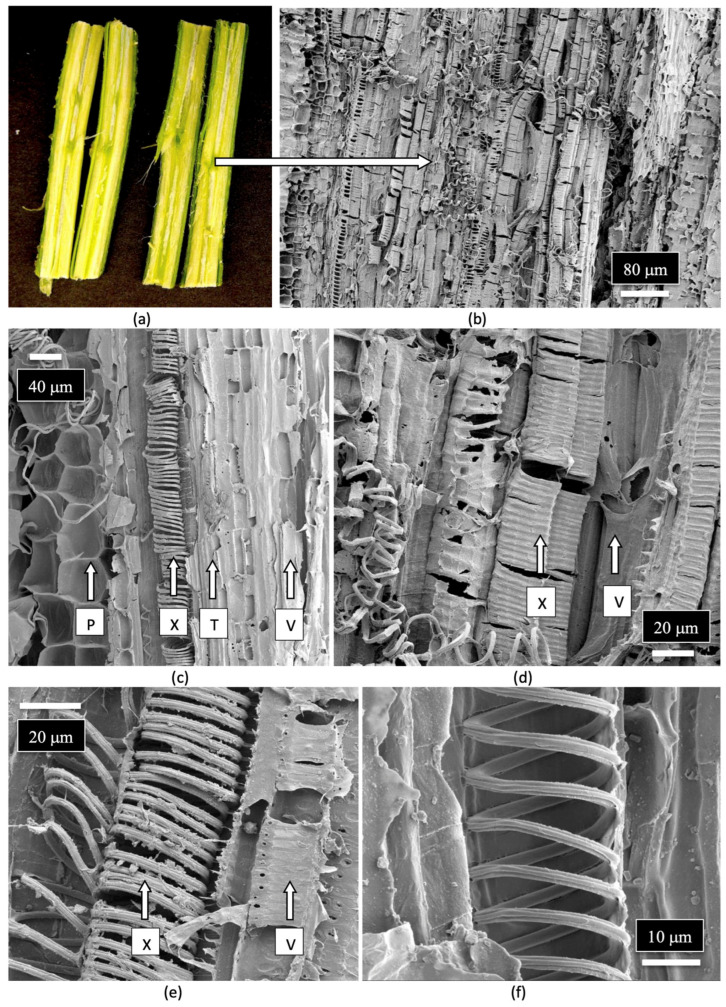
Scanning electron microscopic images of the stem of a cannabis mother plant. (**a**) The stems were cut in longitudinal sections and were examined for the various tissue types as shown in (**b**). (**c**) A closer view of the layers of tissue types from the center of the stem. Moving outwards shows the pith parenchyma cells (P), xylem fibers (X), tracheids (T), and vessel elements (V). (**d**,**e**) A close-up view of the xylem fibers (X) and vessel elements (V). (**f**) A magnified view of the xylem fibers.

When a longitudinal section of the stem of a mother plant (Figure 9a) was examined under the scanning microscope, a complex layer of different tissue types was observed (Figure 9b). At higher magnification, these tissue types were identified as pith parenchyma cells, xylem fiber cells, and longitudinal rows of xylem tracheid cells (Figure 9c). A closer examination also revealed rows of xylem vessel elements interspersed among the xylem fibers (Figure 9d). Magnified views of the xylem fibers and vessel elements are shown in Figure 9e,f.

**Figure 10:**
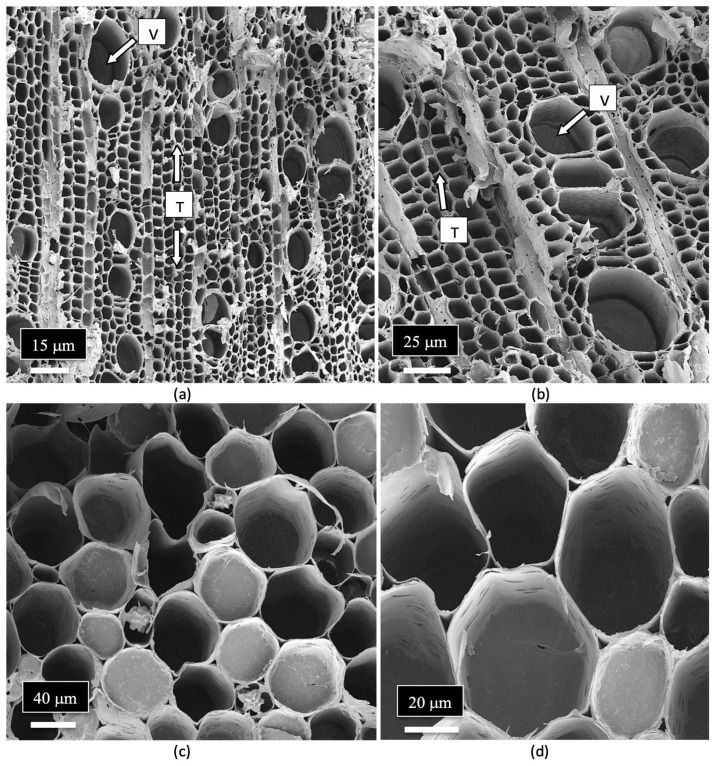
Magnified views of the xylem and pith parenchyma cells found in the stem of a cannabis mother plant as seen under the scanning electron microscope. (**a**) The length-wise arrangement of the tracheids (T) and vessel elements (T) can be seen. (**b**) A higher magnification view of the tracheids (T) and vessel elements (V). (**c**,**d**) A view of the pith parenchyma cells surrounding the pith region.

The arrangement and size of the xylem tracheid cells and vessel elements are shown in Figure 10a,b. Close-up views of the pith parenchyma cells are shown in Figure 10c,d.

**Figure 11:**
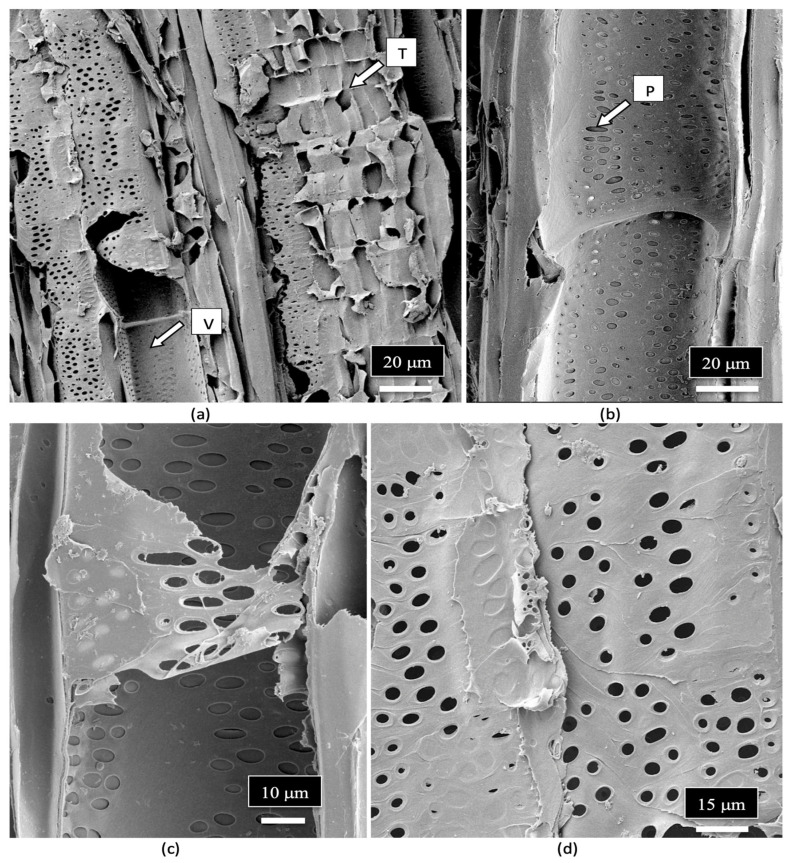
Magnified images of the vessel elements and tracheids found in the stem of a cannabis mother plant as viewed under the scanning electron microscope. (**a**) The longitudinal arrangement and size of the vessel elements (V) and tracheids (T) are shown side-by-side. (**b**) A close-up view of a vessel element cell showing the presence of pit openings (P) along the walls. (**c**,**d**) Magnified views of the pit openings that line the walls of the vessel elements. Pit opening apertures range from 3 μm to 10 μm in diameter.

The vessel elements were surrounded by pitted walls with openings of 3–10 μm in diameter (Figure 11a–d). These pits are essential components in the water transport system of the xylem tissues [110], allowing water to pass between xylem conduits. They are illustrated for the first time in cannabis tissues.

**Figure 12:**
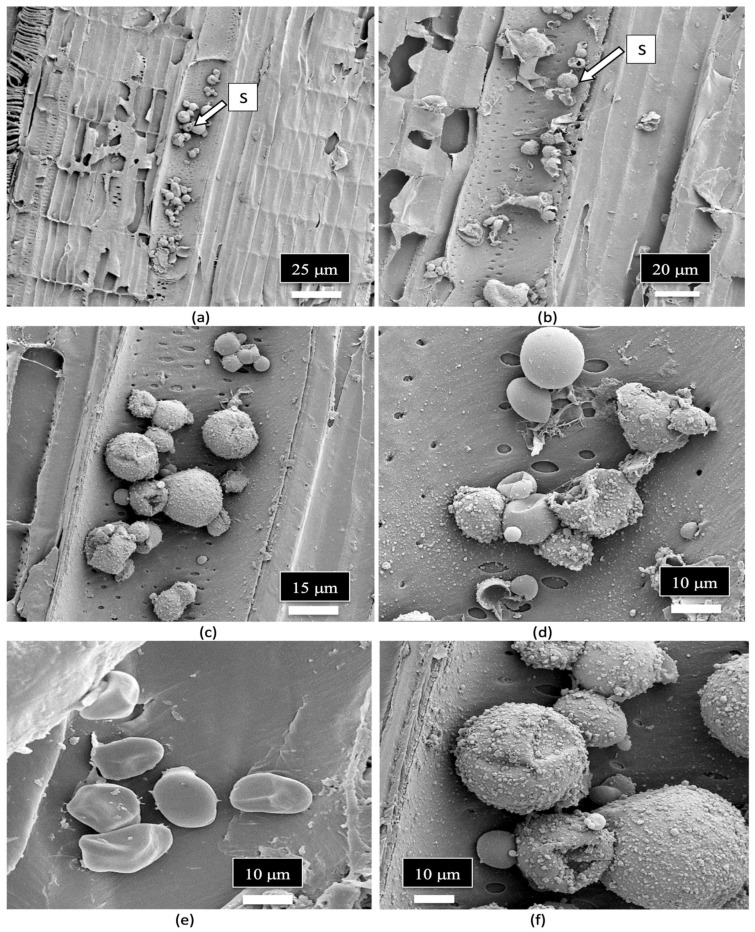
Scanning electron microscopic images of the xylem vessel elements inside the stem of a cannabis mother plant. The surrounding walls of the vessel elements were broken apart to show the presence of fungal spores inside the vessel elements. (**a**,**b**) A range of spore types (S) can be seen lining the vessel element. (**c**,**d**) Close-up views of the mostly spherical spores that represent different sizes, presumably of different fungal species. Some spores appear to be collapsed. (**e**,**f**) Magnified views of different spore types in the xylem vessel elements. Some spores appear to be coated by a biofilm of unknown source.

A close-up examination of the cells of the vessel elements that had broken apart during the sectioning of the stem tissues to reveal the internal structure showed the presence of fungal spores (Figure 12a). When examined under higher magnification, some of the spores appeared to have collapsed (Figure 12b), while others were intact and lined the inside walls of the vessel elements (Figure 12c). The spores were variable in size and shape, reflecting the presence of a number of different species of fungi (Figure 12d,e). Many of the spores were surrounded by what appeared to be a granular material that probably constituted a biofilm (Figure 12f).

**Figure 13:**
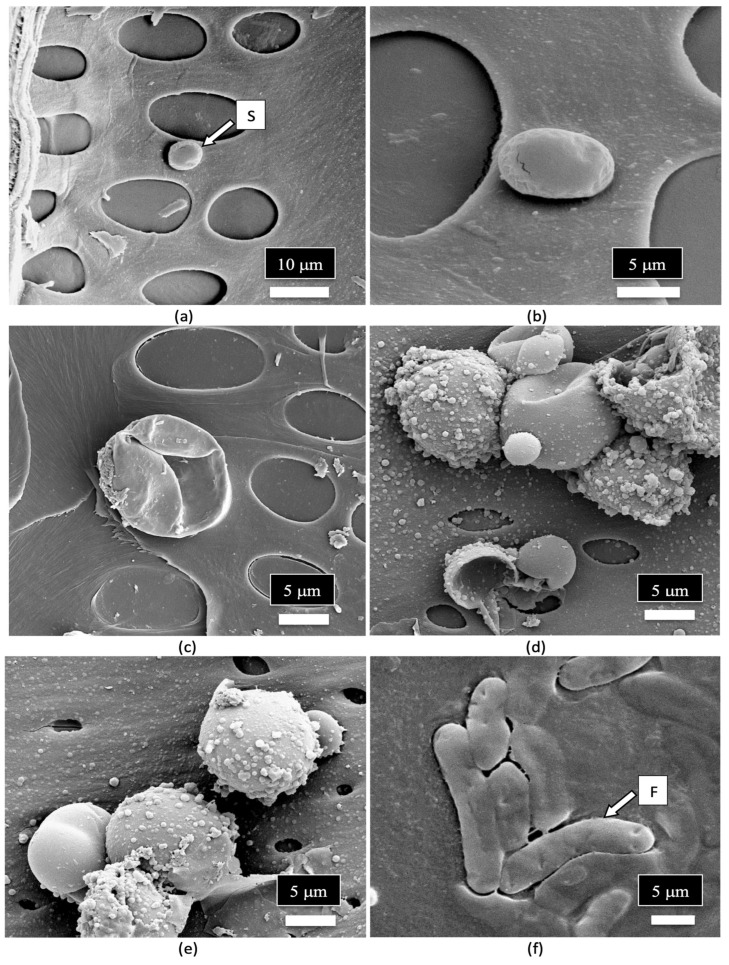
Scanning electron microscopic images of the spores of various fungi in relation to the size of the xylem vessel element pits. (**a**) An extremely small spore that can pass through the pit opening, possibly of *Penicillium.* (**b**) A moderately sized spore that can pass though the pit opening, possibly the microconidia of *Fusarium*. (**c**) A large spore that cannot pass through the pit opening, possibly of *Botrytis*. (**d**,**e**) Very large spores of different fungi that cannot pass through the pit openings, possibly of mycorrhizal fungi. (**f**) Spores resembling *Fusarium* macroconidia at the early stages of development.

A close-up image of fungal spores relative to the size of the pits in the vessel elements is shown in Figure 13. Some of the spores were approximately the size of spores of *Penicillium* or *Aspergillus* and could pass through the pore openings (Figure 13a,b), while other larger spores, such as those of mycorrhizal species, would be transported with the water flow taking place within the vessel elements and were much larger in size (Figure 13c–e). Spores resembling the early developmental stages of the microconidia and macroconidia of *Fusarium* were observed (Figure 13f).

**Figure 14:**
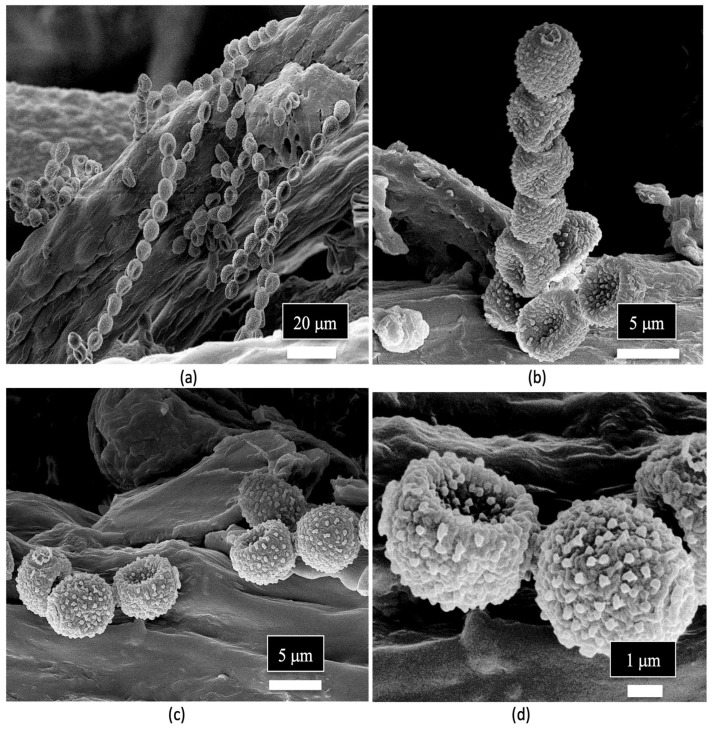
Spores of *Penicillium* sp. observed in the xylem tissues of the stem of a cannabis mother plant. (**a,b**) The spores are produced in distinct chains and (**c,d**) are echinulate (with spines).

Spores of *Penicillium* sp. were observed in the xylem tissues of cannabis stems and frequently formed chains (Figure 14a–d).

**Figure 15:**
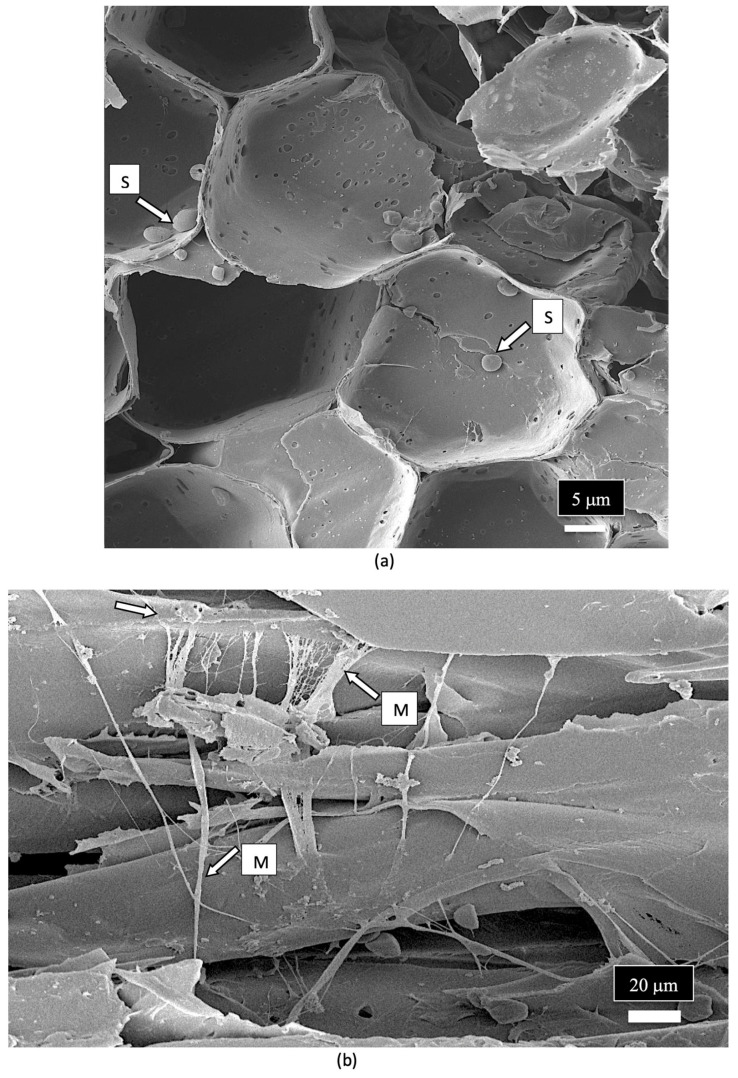
Scanning electron micrograph images of spores and mycelium observed in xylem tissues of the stem of a cannabis mother plant. (**a**) Spores (S, white arrow) are present inside the pith parenchyma cells. (**b**) Mycelium (M, white arrows) can be observed growing over the xylem tissue cells.

The spores of unidentified fungi were also observed in the pith parenchyma cells (Figure 15a). The appearance of mycelial-like structures was observed growing across the pith cells (Figure 15b). Previous scanning electron microscopic studies which have been conducted on other plant species have shown similar internal mycelial colonization of the host by fungi. For example, dormant buds on pecan trees showed internal mycelial growth with hyphal-like strands identical to those seen in cannabis stems [111]. Similarly, mycelial colonization of pine trees by *Phellinus pini* showed hyphal strands in the cells of the xylem tracheids similar to that observed in the present study [112]. Collectively, these studies show that mycelial colonization of xylem tracheids and pith parenchyma cells can occur.

**Figure 16:**
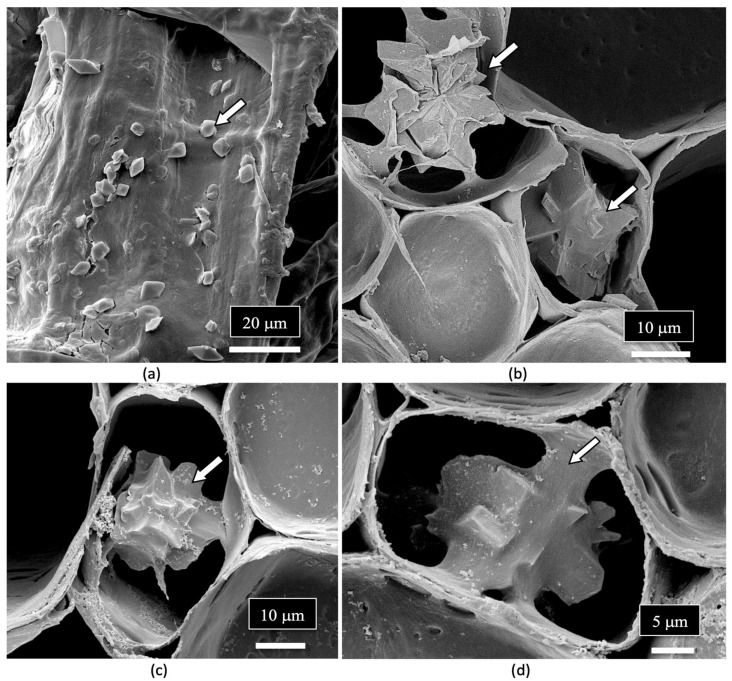
Crystals presumed to be those of calcium oxalate (white arrows) are present in the xylem vessel elements (**a**) as well as in the pith parenchyma cells (**b**–**d**).

Crystals that morphologically resembled those of calcium oxalate were observed lining the vessel elements (Figure 16a) as well as developing inside the pith parenchyma cells (Figure 16b–d). Calcium oxalate crystals are commonly found in many plant species [113], and these crystals have been observed in different plant tissues [114]. These include the mesocarp of tomato fruits [115], parenchyma cells in conifer tree stems [116], xylem vessel lumens, parenchyma cells in the flowers of *Chamelaucium* [117] and in the pith tissues of stems, and near vascular bundles in the leaves of hop plants [114]. Various crystal morphologies resembling those observed in the parenchyma cells of the pith and the xylem vessels of cannabis stem tissues as shown in Figure 16 have been previously reported [114].

**Figure 17:**
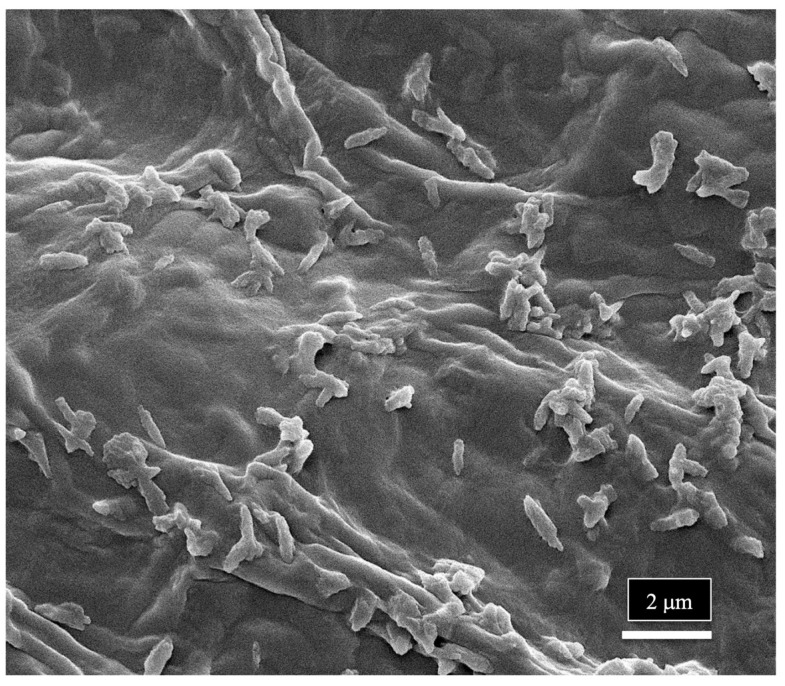
Scanning electron microscopic image showing bacterial cells on the surface of the xylem vessels in a stem sample of cannabis from a mother plant.

The scanning electron microscopic study indicates that a range of spores of endophytic fungi are present in the xylem vessel elements and pith parenchyma cells, where they can be disseminated to other parts of the plant, including the leaves and inflorescences. Their uptake through the roots from the substrate would be facilitated by water flow in the xylem, where they could be transported and become embedded in the xylem tissues. The presence of spores in the xylem cells can explain the large numbers of fungi (up to 20 genera) that are reported to be endophytes in various cannabis tissues. The pith and xylem cells of cannabis plants can therefore harbor these fungi and act as conduits for the movement of these fungi, as well as bacteria, to be distributed in various types of tissues and organs, including seeds. This mechanism could allow bacteria to be disseminated throughout the plant, explaining the large number of reports of recovery of endophytic bacteria from stems, leaves, petioles, flowers, and seeds in previous studies. At the magnifications used in the present study, bacterial cells were not readily discernable; however, in one sample of a cannabis stem from a mother plant, rod-shaped bacterial cells could be seen growing on the sectioned xylem tissues (Figure 17).

It remains to be determined if there is a selective process to how these fungal or bacterial species can invade the xylem and be transported throughout the plant. The ability to traverse any pre-existing defense structures or those induced upon entrance into xylem tissues by potential endophytes could impact their establishment. The ability of fungal pathogens to overcome these defenses is well known, allowing them to become established in the xylem tissues [112], but less is known about the endophytes that are not pathogens. Presumably, the detection of up to 20 genera of fungi from within cannabis tissues suggests that a wide spectrum of fungi, and presumably bacteria, is able to gain access to the internal tissues of cannabis plants and establish in the xylem tissues.

The occurrence of the mycorrhizal species *R. irregularis* in various tissues of the cannabis plant, including stems, leaves, and seeds, which are remote locations from the roots where infection is initiated, is likely the outcome of the transport of the spore types produced within the xylem vessel elements. The large spores shown in Figure 12f could be the resting spores of this species as described in a recent study [92]. This is the first report showing the extensive internal colonization of cannabis tissues by this mycorrhizal species, the significance of which has yet to be established.

**Figure 18:**
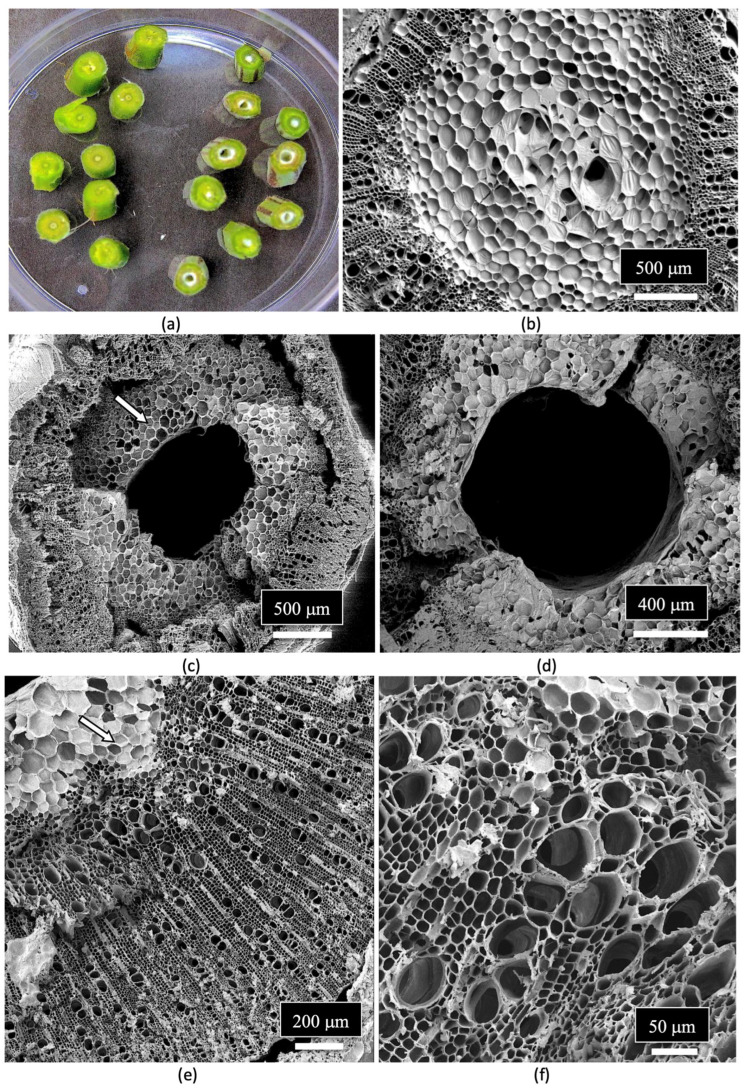
The morphology and tissue types observed in cuttings of cannabis plants under the scanning electron microscope. (**a**) Cross sections made at the base of the cuttings of two genotypes show the different stages of development of the central pith tissues. On the left, the pith in the genotype is less developed compared to the genotype on the right. (**b**) In the genotype from the left side in (**a**), the pith parenchyma cells can be seen in the center of the stem of the cutting, but the pith opening has not yet developed. (**c**,**d**) In the genotype from the right side in (**a**), the pith has fully developed to form an opening, and the pith parenchyma cells are reduced to a ring around the opening (white arrow in (**c**)). (**e**) View of the xylem conducting tissues that comprise tracheids and vessel elements, similar to that seen in the stems of mother plants. The white arrow shows the ring of pith parenchyma cells adjacent to the xylem vessels. (**f**) Close-up view of the tracheids and vessel elements.

The tissue types and arrangement observed in cuttings derived from mother plants of cannabis are shown in Figure 18. A central hollow pith was observed, similar to that seen in the mother plants, but was much larger in size and was surrounded by a ring of pith parenchyma cells that formed a white border (Figure 18a). Under the scanning microscope, the ring of pith parenchyma cells was very distinct and comprised large thin-walled cells (Figure 18b). The large pith opening surrounded by the pith parenchyma cells is shown in Figure 18c,d. Adjacent to that were the thick-walled cells that comprised the tissues of the xylem, namely vessel elements and tracheids, similar to that seen in the stems of the mother plants (Figure 18e,f).

**Figure 19:**
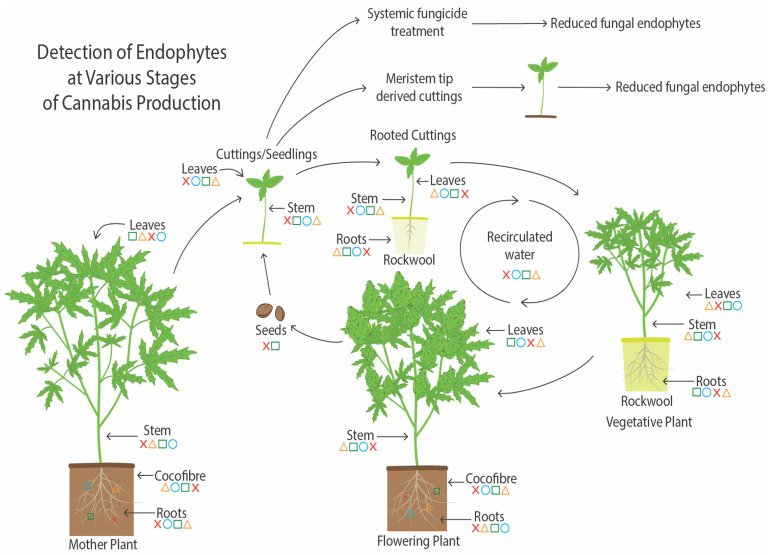
Summary of the distribution of endophytic species of fungi and yeasts during cannabis cultivation under greenhouse conditions. The symbols represent different groups of endophytes that have been found in the various locations based on results presented in this study.

A summary of endophyte presence in cannabis tissues and potential methods for their spread are shown in Figure 19. As demonstrated in this study, the growing substrate and mother plants are a potential source of endophytes, which can be transferred to seeds and cuttings. Vegetative plants derived from cuttings and subsequently flowering plants also contain a proportion of the endophytes present in cuttings. The substrate used to grow the flowering plants can also be a source of endophytes. Additionally, recirculated water may contain and distribute endophytic microbes to the root system. Low levels of endophytes were detected in recirculated water in this study. Reducing the prevalence of endophytic fungi in cannabis cuttings can be achieved through meristem tip culture. Also, the sterilization of the growing substrate followed by the introduction of potentially beneficial endophytic fungi can best be achieved at the rooting stage of cuttings, which was demonstrated in this study following the application of *Trichoderma* spp. to the growing substrate after it was autoclaved. Potential pathogens can also colonize the tissues and establish an endophytic presence at this early stage, particularly in tissue culture-derived plants.

## 8. Future Research

Additional research on the types of endophytes present in cannabis plants is required to establish their functional roles. The widespread presence of certain endophytic species, especially of bacteria, suggests that they may have potential benefits as bio-stimulants and biocontrol agents [21,22]. However, isolating and characterizing these microbes beyond laboratory experiments is essential to confirm their specific functions and interactions with the host plant. In addition, research on understanding the potential negative impacts of endophytic fungi or bacteria on cannabis health and product quality should be considered. The recent literature on cannabis and hemp endophytes is mostly directed towards emphasizing their beneficial roles, deemed in some studies to be essential roles of these endophytes [64,79]. Yet, this is not always the case. The wide range of fungi and yeasts identified through microbiome analyses, culturing, and microscopic observations reported in the present study suggests that many microbes can invade and persist within the xylem and pith cells of cannabis plants. While some may be incidental (accidental) colonizers, others could confer currently undetermined advantages to the plant, yet another subset may be detrimental. This latter aspect has received less attention, with the overwhelming published literature emphasizing endophytes to be of benefit to the plants they inhabit [64,79]. In essence, the conclusions derived seem to be that if microbial species are present inside the plant, they must be performing something good. However, the intricate interactions between endophytes and cannabis and hemp plants, which may lead to beneficial or detrimental effects, are influenced by the microbial species and environmental conditions, which need to be better understood. As stated by Schulz and Boyle [118], “endophytes represent, both as individuals and collectively, a continuum of mostly variable associations: mutualism, commensalism, latent pathogenicity, and exploitation. This may include saprophytes growing on dead or senescent tissues after an endophytic growth phase in the plant, avirulent microorganisms, latent pathogens, virulent pathogens in the early stages of infection, as well as beneficial microbes”. Additional studies are needed to confirm at which point in the spectrum of these interactions the endophytes reported in cannabis and hemp plants may exert beneficial/detrimental effects on the growth and quality of the plants. They cannot all be assumed to provide benefits to the plant by their mere presence.

Our findings highlight two species that are worthy of further study: *Penicillium chrysogenum*, a ubiquitous endophyte with strong antibiotic production capabilities, and the mycorrhizal fungus *Rhizophagus irregularis*, both of which were detected in a wide range of cannabis tissues. Their presence in cannabis plants suggests roles that are not yet fully understood. The reasons for the widespread occurrence of these two fungi, as well as their potential benefits to cannabis growth and quality, warrant further investigation. Future research should focus on determining the environmental and genetic factors that influence endophyte colonization and persistence, as well as identifying strategies to modify the microbiome to enhance plant growth, particularly in the presence of pathogens [74]. Studies are needed to understand the conditions under which endophytes may transition from being mutualistic or commensal to pathogenic. The removal of a large proportion of the endophytic population in cannabis tissues after a cycle of meristem tissue culture demonstrated that these plantlets appeared to be more susceptible to colonization by plant pathogens, such as *Fusarium oxysporum*. In retrospect, this indirectly suggests that a component of the established endophytes may have been providing protection against pathogen invasion, but this needs to be further investigated.

The results presented in this review describe the microbiome composition of hydroponically grown plants under greenhouse conditions. Studies of cannabis plants grown under different conditions and substrates, including organic production, are needed. These studies will likely reveal that a more diverse and complex microbiome is present, with potentially a balance between beneficial and detrimental populations. These studies will help to enhance the development of microbiome-based strategies to optimize cannabis and hemp plant growth. The information presented in this review can provide a foundation upon which further explorations can be based. It is worthy of mention that endophytic microbes selected for practical use to improve cannabis and hemp growth are required to address the regulatory requirements regarding non-target impacts as well as safety issues. The selection of microbes that can successfully navigate these requirements prior to the onset of research projects should help to generate practical results for the cannabis and hemp industries.

## Figures and Tables

**Table 1:** Microbial species recovered as endophytes from various cannabis and hemp plant tissues.

**Reference**	**Wild Cannabis/Field Hemp**	**Indoor Cannabis**	**Reference**
**Seeds**			
[21]	*Bacillus circulans*	*Bacillus aerius*	[21]
	*Bacillus megaterium*	*Bacillus inaquosorum*	
	*Bacillus simplex*	*Bacillus megaterium*	
	*Bacillus subtilis*	*Bacillus simplex*	
	*Bacillus velezensis*	*Bacillus stratosphericus*	
	*Bacillus zhangzhouensis*	*Bacillus subtilis*	
	*Paenibacillus azotifigens*	*Brevibacillus choshinensis*	
	*Paenibacillus humicus*	*Paenibacillus illinoisensis*	
	*Paenibacillus illinoisensis*	*Paenibacillus lautus*	
	*Paenibacillus mobilis*	*Paenibacillus mobilis*	
	*Paenibacillus pabuli*	*Paenibacillus pabuli*	
	*Paenibacillus polymyxa*	*Paenibacillus taohuashanense*	
	*Paenibacillus senegalensis*	*Psychrobacter pulmonis*	
	*Paenibacillus sinopodophylli*	*Niallia circulans*	[25]
	*Paenibacillus taohuashanense*	*Peribacillus frigoritolerans*	
	*Paenibacillus terreus*	*Pseudomonas congelans*	
	*Paenibacillus vulneris*	*Pseudomonas punonensis*	
	*Paenibacillus yunnanensis*	*Rathayibacter festucae*	
	*Pantoea agglomerans*		
[26]	*Bacillus aryabhattai*		
	*Cellulomonas hominis*		
	*Curtobacterium flaccumfaciens*		
	*Kocuria rhizophila*		
	*Paenibacillus amylolyticus*		
	*Psychrobacillus psychrodurans*		
	*Sphingomonas aerolata*		
	*Staphylococcus epidermidis*		
	*Staphylococcus haemolyticus*		
	*Stenotrophomonas rhizophila*		
**Reference**	**Wild Cannabis/Field Hemp**	**Indoor Cannabis**	**Reference**
**Roots**			
[27]	*Acinetobacter gyllenbergii*	*Fusarium oxysporum*	[18]
	*Acinetobacter nosocomialis*	*Fusarium proliferatum*	
	*Acinetobacter oleivorans strain*	*Humicola brevis*	
	*Acinetobacter parvus*	*Humicola fuscoatra*	
	*Acinetobacter pittii*	*Mucor racemosus*	
	*Agrobacterium tumefaciens*	*Trichoderma harzianum*	
	*Bacillus anthracis*	*Afipia felis*	[28]
	*Chryseobacterium kwangjuense*	*Arthrobotrys conoides*	
	*Chryseobacterium vrystaatense*	*Asticcacaulis taihuensis*	
	*Enterobacter asburiae*	*Chaetomium angustispirale*	
	*Enterococcus casseliflavus*	*Fusarium concentricum*	
	*Nocardioides albus*	*Fusarium oxysporum*	
	*Nocardioides kongjuensis*	*Fusarium solani*	
	*Pantoea agglomerans*	*Mesorhizobium opportunistum*	
	*Pantoea vagans*	*Penicillium citrinum*	
	*Planomicrobium chinense*	*Pseudochaetosphaeronema pandanicola*	
	*Pseudomonas putida*	*Trichocladium pyriforme*	
	*Pseudomonas taiwanensis*		
	*Rhizobium radiobacter*		
	*Streptomyces eurocidicus*		
	*Streptomyces werraensis*		
	*Xanthomonas arboricola*		
	*Xanthomonas gardneri*		
**Reference**	**Wild Cannabis/Field Hemp**	**Indoor Cannabis**	**Reference**
**Crowns/Stems**			
[29]	*Alternaria alternata*	*Acremonium alternatum*	[22]
	*Alternaria brassicae*	*Alternaria alternata*	
	*Schizophyllum commune*	*Aspergillus fumigatus*	
[19]	*Alternaria alternata*	*Aspergillus puniceus*	
	*Aspergillus flavus*	*Botrytis cinerea*	
	*Aspergillus nidulans*	*Chaetomium globosum*	
	*Aspergillus niger*	*Cladosporium cladosporioides*	
	*Penicillium chrysogenum*	*Cladosporium globosum*	
	*Penicillium citrinum*	*Lecanicillium aphanocladii*	
	*Rhizopus stolonifer*	*Metarhizium anisopliae*	
		*Chaetomium globosum*	[17]
		*Fusarium oxysporum*	
		*Lecanicillium lanosoniveum*	
		*Penicillium chrysogenum*	
		*Penicillium griseofulvum*	
		*Penicillium olsonii*	
		*Trametes versicolor*	
		*Trichoderma harzianum*	
		*Bacillus licheniformis*	[30]
		*Bacillus megaterium*	
		*Bacillus pumilus*	
		*Bacillus subtilis*	
		*Brevibacillus borstelensis*	
		*Mycobacterium peregrinum*	
		*Penicillium copticola*	[16]
**Reference**	**Wild Cannabis/Field Hemp**	**Indoor Cannabis**	**Reference**
**Leaves/Petioles**			
[31]	*Alternaria alternata*	*Chaetomium globosum*	[16]
	*Aspergillus flavus*	*Eupenicillium rubidurum*	
	*Aspergillus niger*	*Penicillium copticola*	
	*Curvularia lunata*		
	*Penicillium chrysogenum*		
[19]	*Alternaria alternata*		
	*Aspergillus flavus*		
	*Aspergillus niger*		
	*Curvularia lunata*		
	*Penicillium chrysogenum*		
	*Penicillium citrinum*		
**Reference**	**Wild Cannabis/Field Hemp**	**Indoor Cannabis**	**Reference**
**Inflorescences**			
		*Aspergillus versicolor*	[19]
		*Paecilomyces lilacinus*	
		*Penicillium copticola*	
		*Penicillium meleagrinum*	
		*Penicillium sumatrense*	
		*Aspergillus ochraceus*	[32]
		*Penicillium citrinum*	

**Table 2:** Cannabis and hemp bacterial endophytes evaluated for biological control and bio-stimulant properties in vitro or in planta.

Bacterial Species	Effect on Plant or Pathogen *	Mode of Action	Reference
*Bacillus megaterium* and *Brevibacillus borstelensis*	Significantly reduced *Chromobacterium violaceum* production of violacein in cell-free axenic filtrate assays	Quorum quenching	[30]
*Bacillus anthracis* and *Enterobacter asburiae*	Inhibition zone (0.5–1 cm) against *Aspergillus niger* and *Fusarium oxysporum* in dual cultures	Antagonistic mechanisms	[27]
*Pantoea vagans*	Inhibition (0.5 cm) against *Fusarium oxysporum* in dual cultures	Antagonistic mechanisms	[27]
*Pseudomonas taiwanensis* and *Xanthomonas gardneri*	Inhibition (0.5 cm) against *Aspergillus niger* and *Fusarium oxysporum* in dual cultures	Antagonistic mechanisms	[27]
*Pseudomonas putida*, *Comamonas testosteroni*, *Citrobacter freundii*, and *Enterobacter cloacae* added as Mammoth P product	Consortium significantly increased the inflorescence yield (16.5%), plant height (8.9%), and basal stem diameter (13.5%) of hemp	Plant growth promotion mechanisms	[48]
*Serratia plymuthica*	Significantly increased the plant height (11.5%), plant stalk diameter (42%), and plant biomass at harvest (~120%) of field cannabis plants	Plant fitness promotion and biological control	[49]
*Azospirillum brasilense*, *Gluconacetobacter diazotrophicus*, *Burkholderia ambifaria*, and *Herbaspirillum seropedicae*	Consortium significantly increased the stem length (17%), stem dry weight (63%), leaf dry weight (49%), THC (9%), CBN (18%), and CBD (9%) of greenhouse hemp plants	Nitrogen fixation, siderophore production, mineral solubilization, and growth hormone production	[49]
*Pseudomonas fulva* and *P. orientalis*	Significantly inhibited *Sclerotinia sclerotiorum* by 49–53.8% in dual cultures	Antagonistic mechanisms	[20]
*Pseudomonas fulva* and *P. orientalis*	Significantly inhibited *Botrytis cinerea* by 22–18.6% in dual cultures	Antagonistic mechanisms	[20]
*Pseudomonas orientalis*	Significantly inhibited *Rhizoctonia solani* by 27.6% in dual cultures	Antagonistic mechanisms	[20]
*Bacillus subtilis*	Significantly increased dried vegetative biomass of cannabis plants by ~57.1%	Plant growth promotion mechanisms	[54]
*Serratia marcescens*, *Enterobacter cloacae*, and *Paenibacillus hunanensis*	Significantly inhibited *Phytophthora parasitica* by up to 89.6–93.8% in detached cannabis leaf assays	Antibiosis (antifungal metabolite production)	[55]
*Gluconacetobacter diazotrophicus*, *Burkholderia ambifaria*, and *Herbaspirillum seropedicae*	Significantly inhibited *Fusarium oxysporum* by 64–68% in dual cultures	Antibiosis (antifungal metabolite production)	[56]
*Azospirillum brasilense*, *Gluconacetobacter diazotrophicus*, *Burkholderia ambifaria*, and *Herbaspirillum seropedicae*	Consortium significantly inhibited *Fusarium oxysporum* by 70.6% in dual cultures	Antibiosis (antifungal metabolite production)	[56]
*Azospirillum brasilense*, *Gluconacetobacter diazotrophicus*, *Burkholderia ambifaria*, and *Herbaspirillum seropedicae*	Consortium significantly reduced *Fusarium oxysporum* damage by 65%, increased germination of seeds (79%), and increased the development of the roots (86%), shoots (152%), and leaves (133%) of infected greenhouse hemp plants	Antibiosis (antifungal metabolite production), induced systemic resistance, phytohormone production, nutrient solubilization, and nitrogen fixation	[56]
*Bacillus velezensis*, *Bacillus subtilis*, *Pseudomonas synxantha*, and *Pseudomonas protegens*	Inhibited the following fungal pathogens by ~25–75%: *Botrytis cinerea*, *Sclerotinia sclerotiorum*, *Fusarium oxysporum*, *F. culmorum*, *F. sporotrichioides*, *Nigrospora oryzae*, *N. sphaerica*, and *Alternaria alternata* in dual culture assays	Antibiosis (antifungal metabolite production)	[57]
*Bacillus velezensis*, *Bacillus subtilis*, and *Pseudomonas protegens*	Significantly inhibited *B. cinerea* by 25–56% in detached cannabis leaf assays	Antibiosis (antifungal metabolite production)	[57]
*Bacillus inaquosorum*, *Paenibacillus polymyxa*, *Bacillus subtilis*, and *Bacillus velezensis*	Variably inhibited *Alternaria destruens*, *Aspergillus fumigatus*, *Fusarium fujikuroi*, and *Penicillium lanosocoeruleum* (depending on the species and strain) in dual culture assays	Antagonistic mechanisms	[21]
*Bacillus amyloliquefaciens* added as Stargus	Significantly reduced *Fusarium oxysporum* disease severity values of cannabis plants	Antagonistic mechanisms	[22]
*Sphingomonas areolate* and *Chlorella* sp. (*algae*)	Consortium significantly increased the sprout length (11%), root length (~19%), and shoot length (~5%) of hemp plants in vivo	Phytochemical production stimulation and phytohormone production	[58]
*Bacillus frigoritolerans*	Significantly increased the plant height (43.3%), plant stalk diameter (96%), and plant biomass at harvest (~260%) of field cannabis plants	Phytohormone production, bio-fertilization, immune response stimulation, and competitive exclusion	[25]
*Bacillus velezensis*	Significantly inhibited *Agroathelia rolfsii* by 80.5% in dual culture assays and 74.1% in greenhouse hemp plants	Antibiosis (antifungal metabolite production, volatile organic compounds)	[59]

* Efficacy percentages presented are averages derived from published data, including visual approximations from graphical representations in some instances.

**Table 3:** Cannabis and hemp fungal endophytes evaluated for biological control and bio-stimulant properties in vitro or in planta.

Fungal Species	Effect on Plant or Pathogen *	Mode of Action	Reference
*Aspergillus versicolor*, *Chaetomium globosum*, *Eupenicillium rubidurum*, *Paecilomyces lilacinus*, *Penicillium copticola*, *Penicillium meleagrinum*, and *Penicillium sumatrense*	Inhibited *Botrytis cinerea* by ~15.8–100% depending on the biological control agent species, strain, and media used in dual culture assays	Antagonistic mechanisms	[16]
*Trichoderma harzianum*	Significantly increased the plant height (9.65%), dry biomass weight (12.83%), and root density (13.72%) of greenhouse hemp plants	Plant growth promotion (nutrient capture and solubilization)	[60]
*Rhizophagus aggregatus*	Significantly increased the plant height (27.5%), stem diameter (5.8%), leaf area (50.1%), number of branches (53.1%), number of inflorescences (56.5%), and leaf dry weight (50%), root dry weight (63.8%) of laboratory cannabis plants, compared to fertilized control plants	Nutrient and water capture and induced systemic resistance	[61]
*Rhizophagus irregularis*, *Trichoderma harzianum*, *Dictyosphaerium chlorelloides* (*algae*), and *Bacillus subtilis* (*bacteria*) added as Ferticann	Variably affected (cultivar dependent) CBDV, CBG, CBD, CBDA, CBGA, CBN, ∆9-THCA-A, CBNA, CBLA, CBC, CBCA, and flower biomass of laboratory cannabis plants	Production of plant growth compounds and secondary metabolites	[28]
*Gliocladium catenulatum* added as Lalstop, *Trichoderma harzianum* and *Trichoderma virens* added as Rootshield Plus, and *Trichoderma asperellum* added as Asperello	Significantly reduced *Fusarium oxysporum* disease severity values of cannabis plants	Antibiosis, mycoparasitism, competitive exclusion, and induced systemic resistance	[22]
*Gliocladium catenulatum* added as Lalstop	Significantly reduced *Pythium myriotylum* disease severity values of cannabis plants	Antibiosis, mycoparasitism, and induced systemic resistance	[22]

* Efficacy percentages presented are averages derived from published data, including visual approximations from graphical representations in some instances.
